# The microneme adhesive repeat domain of MIC3 protein determined the site specificity of *Eimeria acervulina, Eimeria maxima*, and *Eimeria mitis*

**DOI:** 10.3389/fimmu.2023.1291379

**Published:** 2023-11-08

**Authors:** Yang Zhang, Mingmin Lu, Zhenchao Zhang, Xinmei Huang, Jingwei Huang, Jiabin Liu, Jianmei Huang, Xiaokai Song, Lixin Xu, Ruofeng Yan, Xiangrui Li

**Affiliations:** The Ministry of Education Joint International Research Laboratory of Animal Health and Food Safety, College of Veterinary Medicine, Nanjing Agricultural University, Nanjing, China

**Keywords:** MIC3 protein, MAR, site specificity, *Eimeria*, chicken

## Abstract

Understanding the determinants of host and tissue tropisms among parasites of veterinary and medical importance has long posed a substantial challenge. Among the seven species of *Eimeria* known to parasitize the chicken intestine, a wide variation in tissue tropisms has been observed. Prior research suggested that microneme protein (MIC) composed of microneme adhesive repeat (MAR) domain responsible for initial host cell recognition and attachment likely dictated the tissue tropism of *Eimeria* parasites. This study aimed to explore the roles of MICs and their associated MARs in conferring site-specific development of *E. acervuline*, *E. maxima*, and *E. mitis* within the host. Immunofluorescence assays revealed that MIC3 of *E. acervuline* (EaMIC3), MIC3 of *E. maxima* (EmMIC3), MIC3 of *E. mitis* (EmiMIC3), MAR3 of EaMIC3 (EaMIC3-MAR3), MAR2 of EmMIC3 (EmMIC3-MAR2), and MAR4 of EmiMIC3 (EmiMIC3-MAR4), exhibited binding capabilities to the specific intestinal tract where these parasites infect. In contrast, the invasion of sporozoites into host intestinal cells could be significantly inhibited by antibodies targeting EaMIC3, EmMIC3, EmiMIC3, EaMIC3-MAR3, EmMIC3-MAR2, and EmiMIC3-MAR4. Substitution experiments involving MAR domains highlighted the crucial roles of EaMIC3-MAR3, EmMIC3-MAR2, and EmiMIC3-MAR4 in governing interactions with host ligands. Furthermore, animal experiments substantiated the significant contribution of EmiMIC3, EmiMIC3-MAR4, and their polyclonal antibodies in conferring protective immunity to *Eimeria*-affiliated birds. In summary, EaMIC3, EmMIC3, and EmiMIC3 are the underlying factors behind the diverse tissue tropisms exhibited by *E. acervuline*, *E. maxima*, and *E. mitis*, and EaMIC3-MAR3, EmMIC3-MAR2, and EmiMIC3-MAR4 are the major determinants of MIC-mediated tissue tropism of each parasite. The results illuminated the molecular basis of the modes of action of *Eimeria* MICs, thereby facilitating an understanding and rationalization of the marked differences in tissue tropisms among *E. acervuline*, *E. maxima*, and *E. mitis*.

## Introduction

Avian coccidiosis, a consequence of *Eimeria* infections, is renowned for its high morbidity and mortality, with significant implications for poultry health and welfare (1, 2). The primary bird species affected by *Eimeria* include chickens (both in layer and broiler systems), turkeys, and fowls. Globally, the annual expenditure for the treatment and control of chicken coccidiosis is estimated to be up to $14.5 billion (3). Seven species of *Eimeria*, namely *E. tenella*, *E. acervulina*, *E. mitis*, *E. brunetti*, *E. praecox*, *E. necatrix*, and *E. maxima*, are recognized to infect chickens, each differing in pathogenicity (4). *E. tenella*, *E. acervulina*, *E. necatrix*, and *E. maxima* are highly pathogenic, while *E. mitis* and *E. praecox* are less infectious (5). The life cycle of *Eimeria* parasites comprises two distinct phases: the exogenous phase and the endogenous phase (1). Once ingested by chickens, the oocysts release sporozoites, the initial infective units specialized for invading host intestinal cells (6, 7). The endogenous reproductive stage commences with schizogony (asexual reproduction), followed by gametogony (sexual differentiation) (5). In the schizogony stage, free sporozoites replicate within host cells to form schizonts, while in the gametogony stage, the final generation of mature merozoites differentiates into gametes, which unite to generate zygotes, ultimately maturing into oocysts (6, 7).

Host and tissue tropisms are the ability of a given parasite to preferentially target a particular host or tissue, and the study of host and tissue tropisms has been a central topic for our understanding of parasitology as well as parasitic diseases. Although the phenomenon of aforementioned tropisms has long been observed from a variety of parasites of veterinary and medical significance, including helminths and protozoa, the general mechanism description of parasitic tropisms has posed a grand challenge for decades. For example, the coccidian parasite *Toxoplasma gondii* could infect almost each of warm-blooded vertebrates (8, 9), whereas *Eimeria* parasites within the same coccidia sub-class are individually highly host-specific (9). Moreover, the parasites that belong to the same *Eimeria* genus even display widely varying tissue tropisms, as demonstrated by chicken coccidia spp. These obligatory intracellular parasites are recognized to vary in site-specificity of development in the intestinal tract of birds. *E. acervulina* and *E. praecox* parasitize in the upper intestine, and *E. maxima* and *E. necatrix* develop in the mid intestine. In stark contrast, *E. brunetti* and *E. mitis* infected cells of the lower intestine, and *E. tenella* infected cells of the ceca (10) However, the molecular basis for tissue tropisms of these *Eimeria* parasites manifested long-term unresolved mysteries in the disease pathogenesis of avian coccidiosis.

Generally, the mechanisms of *Eimeria* to invade the chicken intestinal epithelial cells have been considered identical to the cell entry machinery of other apicomplexans (11, 12). Coccidia initially glided over the surface of a host cell and then reoriented to place its apical end in close contact with the host cell membrane. After initial attachment, a circumferential ring of adhesion (called the moving or tight junction) was formed, through which the parasite actively propelled itself while concurrently depressing the host-cell membrane to create a nascent protective vacuole (13). Of particular significance in this context is the adhesion process, which allows the further establishment of forced rapid invasion of host cells by *Eimeria* parasites. Secretory organelles, including micronemes and rhoptries, have been described to release a spectrum of proteins to help *Eimeria* parasites adhere to the host cell (14). Beyond that, micronemes proteins (MICs) also contribute to the invasion machinery by complexing with rhoptry proteins, and the complexes were later targeted into host cells.

To date, a spectrum of MICs of *Eimeria* spp. of chicken was identified and characterized, including ten of *E. tenella* (EtMIC1-7 and EtAMA1-3) (15–22), seven of *E. acervulina* (EaMIC2, EaMIC3, EaMIC5, EaMIC7, EaMIC13, EaMIC14 and EaAMA1 (23–25), six of *E. maxima* (EmMIC1, EmMIC2, EmMIC3, EmTFP250, EmMIC5, EmMIC7, and EmAMA1) (26–31), five of *E. mitis* (EmiMIC2, EmiMIC3, EmiMIC7, EmiAMA1, and EmiEtmic-2/7h) (32), six of *E. necatrx* (EnMIC2, EnMIC3, EnMIC5, EnMIC7, EnMIC13, EnAMA1) and two of *E. brunetti* (EbMIC2 and EbAMA1) (33). Although their functional roles in host-parasite interactions have not been fully elucidated, a few of these MICs have been reported to allow *Eimeria* spp. to bind a diverse range of oligosaccharide epitopes of host cells to drive parasite invasion (34, 35). For instance, MIC3 of *E. tenella* (EtMIC3), through the recognition of sialyl glycans, could guide the parasite to the site of invasion in the ceca.

Like MICs of *Neospora caninum* and *Toxoplasma gondii*, *Eimeria* MICs also bear the microneme adhesive repeat domain (MAR). All apicomplexan MARs were classified into Types I and II domains based on their subtle structural differences. The latter has an additional C-terminal β-finger chain, while Type I MARs have an extended α-loop chain. Both Types I and II MARs have been identified in *Toxoplasma* and *Plasmodium* MICs. However, *Eimeria* MICs appear to possess only Type I domains, which may be associated with the host and site specificity of *Eimeria* parasites. For example, MIC3 of *E. mitis* (EmiMIC3) contained nine Type I MARs (MARs1-9), and EtMIC3 consists of seven Type I MAR domains, namely, MAR1a, MAR1b, three repeated MAR1c, MAR1d, and MAR1e (36). Identically, Type I MAR domains of *Eimeria* MICs mediate parasite recognition of host cell molecules. Their sequence divergence alongside structural differences of MAR domains may account for differential binding properties. For *E. tenella*, there is considerable variability in the binding capability of all MARs of EtMIC3 to cell surface sialyl glycans, while MAR1b exhibits more robust binding signals than MAR1a, MAR1c, MAR1d, and MAR1e and may govern tissue tropisms of *E. tenella* (37, 38).

Sequence alignment analysis showed that two common LxxY and HxT/HxS motifs were shared by all MARs of EtMIC3, which were responsible for the specific binding of EtMIC3 protein to sialyl acid saccharide on host cells and may help *E. tenella* target chicken cecal epithelial cells. However, this is not the case for other *Eimeria* parasites. Specifically, the LxxY motif was presented in the MAR1, MAR2, MAR3 and MAR8 domains of EmiMIC3, whereas the motif of HxT/HxS was identified in MAR2, MAR3, MAR4, MAR5, MAR6, and MAR7 domains of EmiMIC3 (32). The predominant sequence difference in MARs of EtMIC3 and EmiMIC3 suggested that the key factors or the molecules that governed the site specificities of *Eimeria* spp. (parasitizing in the small intestine or the ceca) might be completely different. The tissue tropism machinery of coccidia parasites goes beyond the general conceptual framework of the apicomplexan invasion process and includes the binding specificity of *Eimeria* MICs, as well as their unique Type I MARs. Although recent studies have revealed the engagement of EtMICs in the invasion machinery of *E. tenella*, the diversified roles of EmiMICs, MICs of *E. maxima* (EmMICs), and MICs of *E. acervuline* (EaMICs) remain largely unknown. As such, the objective of this study was to identify the roles of EmiMICs, EmMICs, and EaMICs, alongside their unique MARs in the site specificities of *E. acervulina*, *E. maxima*, and *E. mitis*.

## Results

### EaMIC3, EmMIC3 and EmiMIC3 selectively bind to the upper, mid and lower intestines

**Figure 1:**
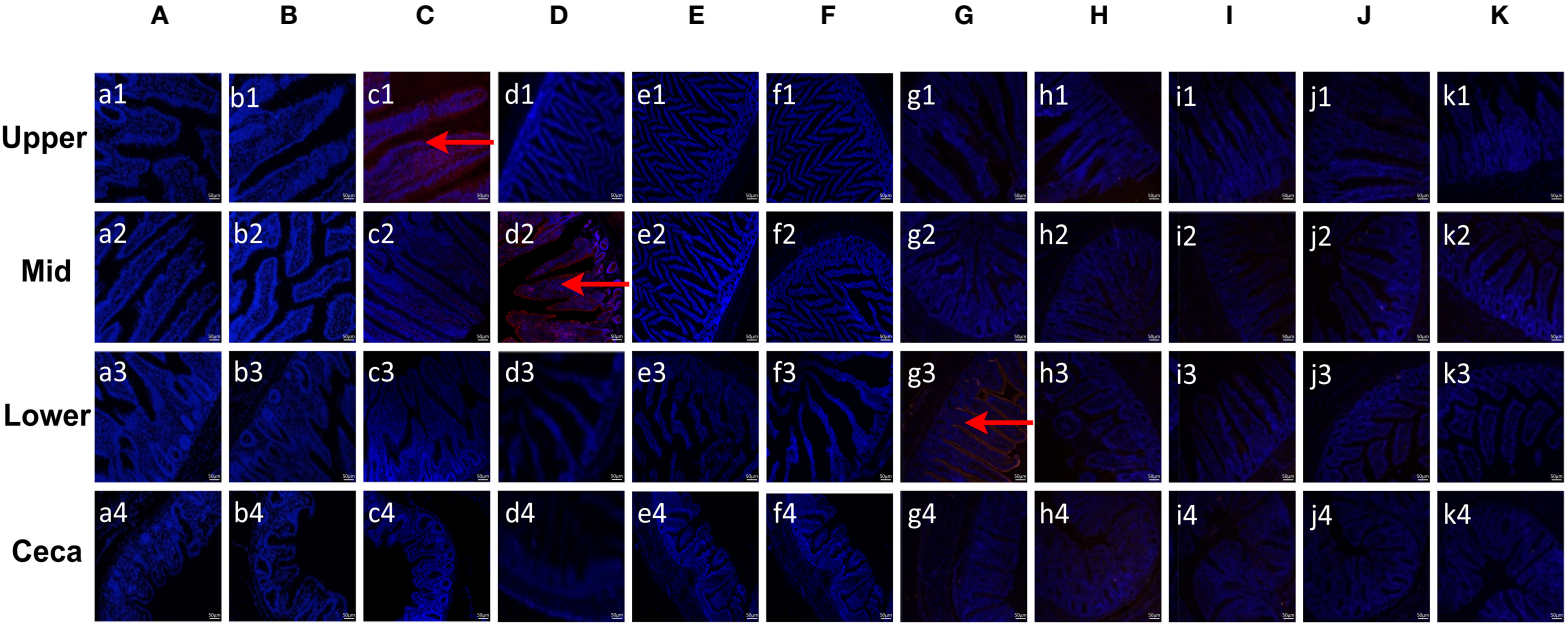
Binding properties of *Eimera* microneme proteins to different small intestine segments. The upper, mid, lower intestine, and caecum tissues were removed from 2-week-old chicken. The substantial red fluorescence from labeling EaMIC3 **(C)** was observed in sections of the upper intestines, red fluorescence resulting from tagging EmMIC3 **(D)** with anti-EmMIC3 pAbs, and the red fluorescence from labeling EmiMIC3 **(G)** was observed in sections of the lower intestines, while EaMIC2 **(A)**, EaMIC5 **(B)**, EmMIC2 **(E)**, EmAMA1 **(F)**, EmiMIC2 **(H)**, EmiAMA1 **(I)**, EmiEtmic-2/7 **(J)**, and pET-32a **(K)**, no red fluorescence was identified. Scale bars: 50 μm.

To evaluate the binding specificity of different EaMICs, EmMICs, and EmiMICs to host tissues, intestinal samples were taken from throughout the chicken gut (the upper, mid, and lower intestine, and caecum), and immunofluorescence staining of EaMICs, EmMICs, and EmiMICs with specific anti-EaMICs, anti-EmMICs, and anti-EmiMICs antibodies was performed respectively. As shown in **Figure 1C**, substantial red fluorescence from labeling EaMIC3 was observed in sections of the upper intestines (**Figure 1**, c1) but not those of the mid and lower intestines and caecum. In addition, we did not observe any red fluorescence from either EaMIC2-treated (**Figure 1A**) or EaMIC5-treated (**Figure 1B**) histological sections taken from the upper, mid, and lower intestines and caecum, which indicated that EaMIC3 but not EaMIC2 or EaMIC5 could bind to the upper intestine and may play a key role in directing *E. acervulina* to this specific location.

Moreover, the tissue staining pattern was also investigated for EmMIC3 (**Figure 1D**), EmMIC2 (**Figure 1E**), and EmAMA1 (**Figure 1F**). In sections taken from the mid intestines, there was intense red fluorescence resulting from tagging EmMIC3 with anti-EmMIC3 pAbs (**Figure 1**, d2), suggesting that EmMIC3 could remain in close contact with host epithelial cell derived from the mid intestines. However, no red fluorescence was identified in rEmMIC3-treated sections of the upper and lower intestines and caecum (**Figure 1**, d1, d3, d4). As for EmMIC2 and EmAMA1, no red fluorescence was detected from any EmMIC2-treated or EmAMA1-treated gut sections (**Figures 1E, F**). In addition, incubation of these gut sections with rEmiMIC3 (**Figure 1G**), rEmiMIC2 (**Figure 1H**), rEmiAMA1 (**Figure 1I**), rEmiEtmic-2/7 (**Figure 1J**) showed an abundance of EmiMIC3 staining in sections taken from the lower intestines (**Figure 1**, g3). The absence of binding signals of EmiMIC2, EmiAMA1, and EmiEtmic-2/7 to the section derived from throughout the chicken gut suggested a specific binding pattern for EmiMIC3, and the binding was predominantly to the lower intestines.

### The MARs of EaMIC3, EmMIC3, and EmiMIC3 have relatively conserved binding domains

**Figure 2:**
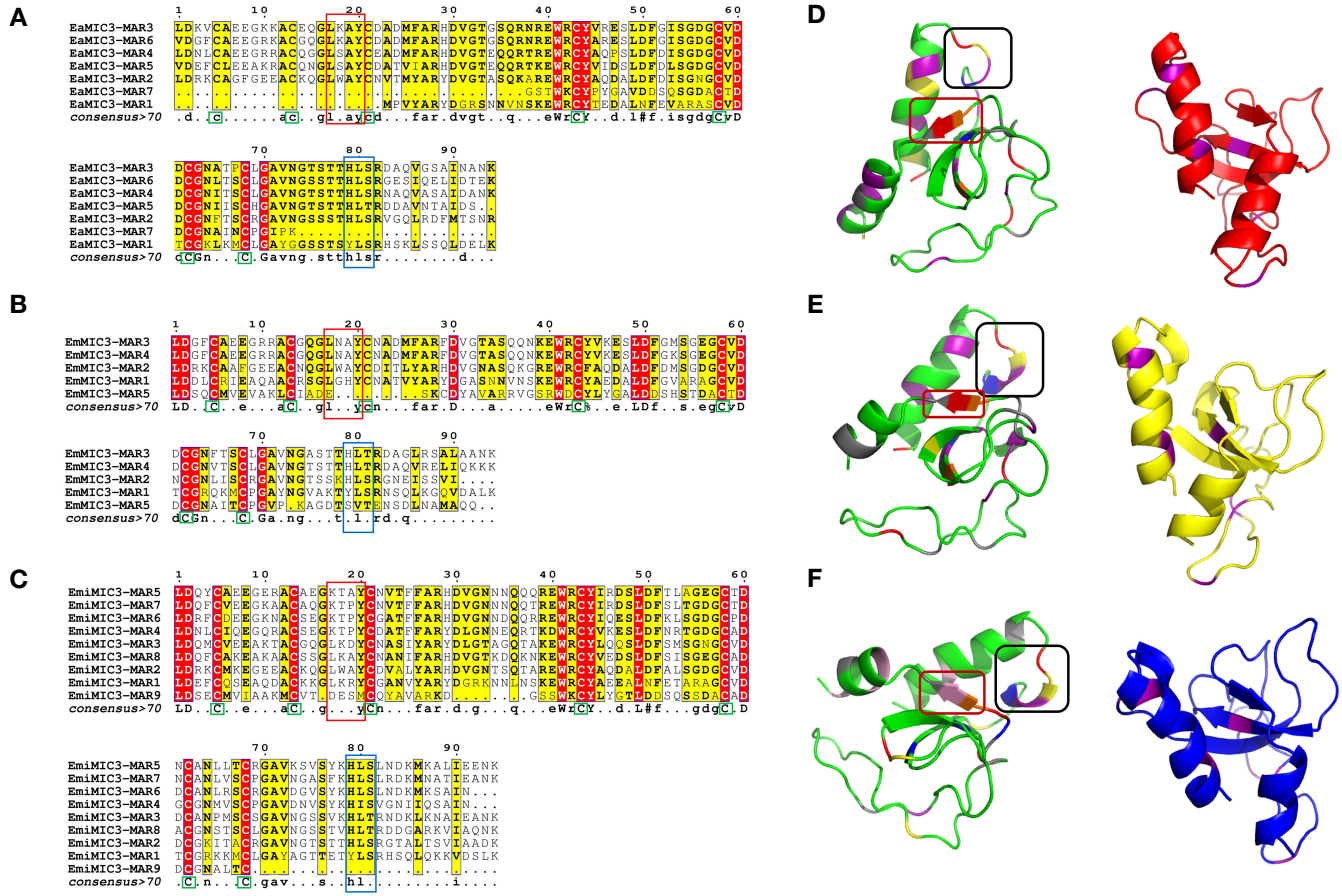
Alignment of MAR domains and the three-dimensional structure of the EaMIC3-MAR3, EmMIC3-MAR2, and EmiMIC3-MAR4. MAR domains from EaMIC3 (7 repeats) **(A)**, MAR domains from EmMIC (5 repeats) **(B)**, MAR domains from EmiMIC3 (9 repeats) **(C)**. The high similarity is represented by different intensities of red/yellow, respectively. Green boxes indicate conserved cysteines **(C)**, red boxes indicate LxxY residue with the α1-helix/loop extension of MAR type I; blue boxes indicate HxT/HxS residue coordinating binding to the sialic acid saccharides. The left structures of EaMIC3-MAR3 **(D)**, EmMIC3-MAR2 **(E)**, EmiMIC3-MAR4 **(F)** represent the spatial distribution of the LxxY and HxS/HXT motif, and the black frame indicates the LxxY motif and the red border indicates the HxS/HXT motif. The right images represent the cysteine distribution region, and the purple sections are cysteine.

Given that EaMIC3, EmMIC3, and EmiMIC3 might be responsible for guiding *E. maxima*, *E. acervulina*, and *E. mitis* to corresponding invasion sites in the chicken gut, respectively, we further aligned MARs from EaMIC3, EmMIC3, and EmiMIC3 so as to predict their potential binding properties. The EaMIC3-MAR1 and EaMIC3-MAR7 of EaMIC3, EmMIC3-MAR5 of EmMIC3, and EmiMIC3-MAR9 of EmiMIC3 were incomplete repeating sequences. Most of the EaMIC3-MARs, EmMIC3-MARs, and EmiMIC3-MARs contain seven cysteine (C) residues (**Figures 2A–C**, green boxes) except for EaMIC3-MAR1 and EaMIC3-MAR7 which lack the first three C residues and EmMIC3-MAR5 which lack the third one. Most MARs from EaMIC3, EmMIC3, and EmiMIC3 exhibit the conserved signatures of Type I MAR of *Eimeria* parasites. The LxxY motif within the α1-helix/loop extension of Type I MAR identified in EaMIC3-MAR2, EaMIC3-MAR3, EaMIC3-MAR4, EaMIC3-MAR5 and EaMIC3-MAR6 of EaMIC3, EmMIC3-MAR1, EmMIC3-MAR2, EmMIC3-MAR3 and EmMIC3-MAR4 of EmMIC3, and EmiMIC3-MAR1, EmiMIC3-MAR2, EmiMIC3-MAR3 and EmiMIC3-MAR8 of EmiMIC3 were relatively conserved (**Figures 2A–C**, red boxes). Moreover, the HxT/HxS signature that directly coordinates binding to sialyl saccharides found in EaMIC3-MAR2, EaMIC3-MAR3, EaMIC3-MAR4, EaMIC3-MAR5 and EaMIC3-MAR6 of EaMIC3, EmMIC3-MAR2, EmMIC3-MAR3 and EmMIC3-MAR4 of EmMIC3, and EmiMIC3-MAR2, EmiMIC3-MAR3, EmiMIC3-MAR4, EmiMIC3-MAR5, EmiMIC3-MAR6, EmiMIC3-MAR7 and EmiMIC3-MAR8 of EmiMIC3 were also considerably conserved (**Figures 2A–C**, blue boxes). The results from multiple sequence alignment suggested that most MARs from EaMIC3, EmMIC3, and EmiMIC3 have pretty high conservation in their binding motifs.

### EaMIC3-MAR3, EmMIC3-MAR2, and EmiMIC3-MAR4 exerted strong binding abilities to differing intestinal tissues

**Figure 3:**
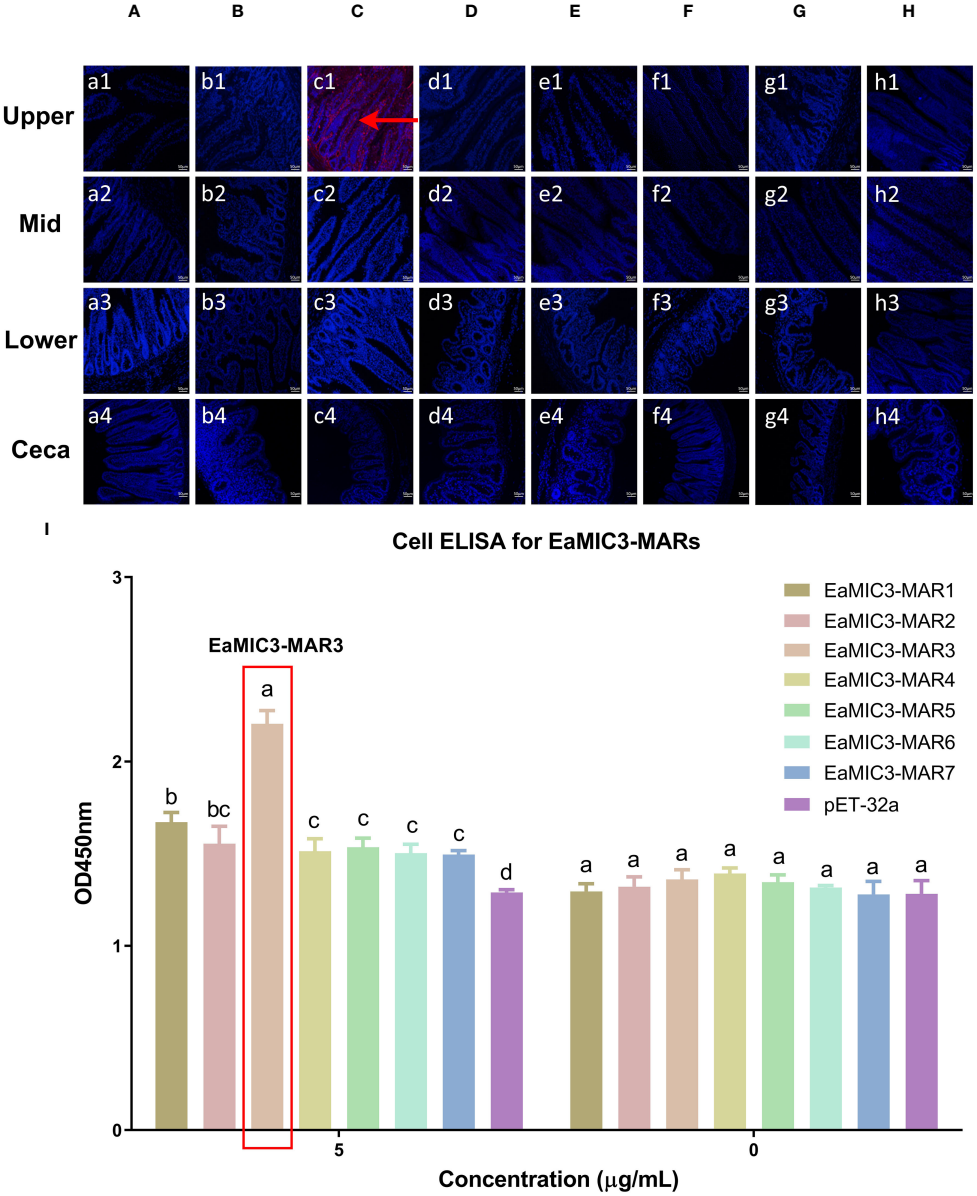
Binding properties of EaMIC3-MARs to different intestine segments. The upper, mid, lower intestine, and caecum tissues were removed from 2-week-old chicken. EaMIC3-MAR3 **(C)** gave the most robust binding to the upper intestine sections (red fluorescence), while EaMIC3-MAR1 **(A)**, EaMIC3-MAR2 **(B)**, EaMIC3-MAR4 **(D)**, EaMIC3-MAR5 **(E)**, EaMIC3-MAR6 **(F)**, EaMIC3-MAR7 **(G)**, and pET-32a **(H)** did not show significant binding to intestinal tissue. Scale bars: 50 μm. The upper intestinal epithelial cells were isolated and cultivated from 2-week-old chickens. The chicken upper epithelial cells were co-cultured respectively with the recombinant EaMIC3-MARs (0 μg/mL or 5 μg/mL). EaMIC3-MAR3 exhibited the most robust binding signals to the upper epithelial cells in a dose-dependent manner **(I)**. Among different MARs, the significant difference within the group was shown by different letters (*p* < 0.05).

**Figure 4:**
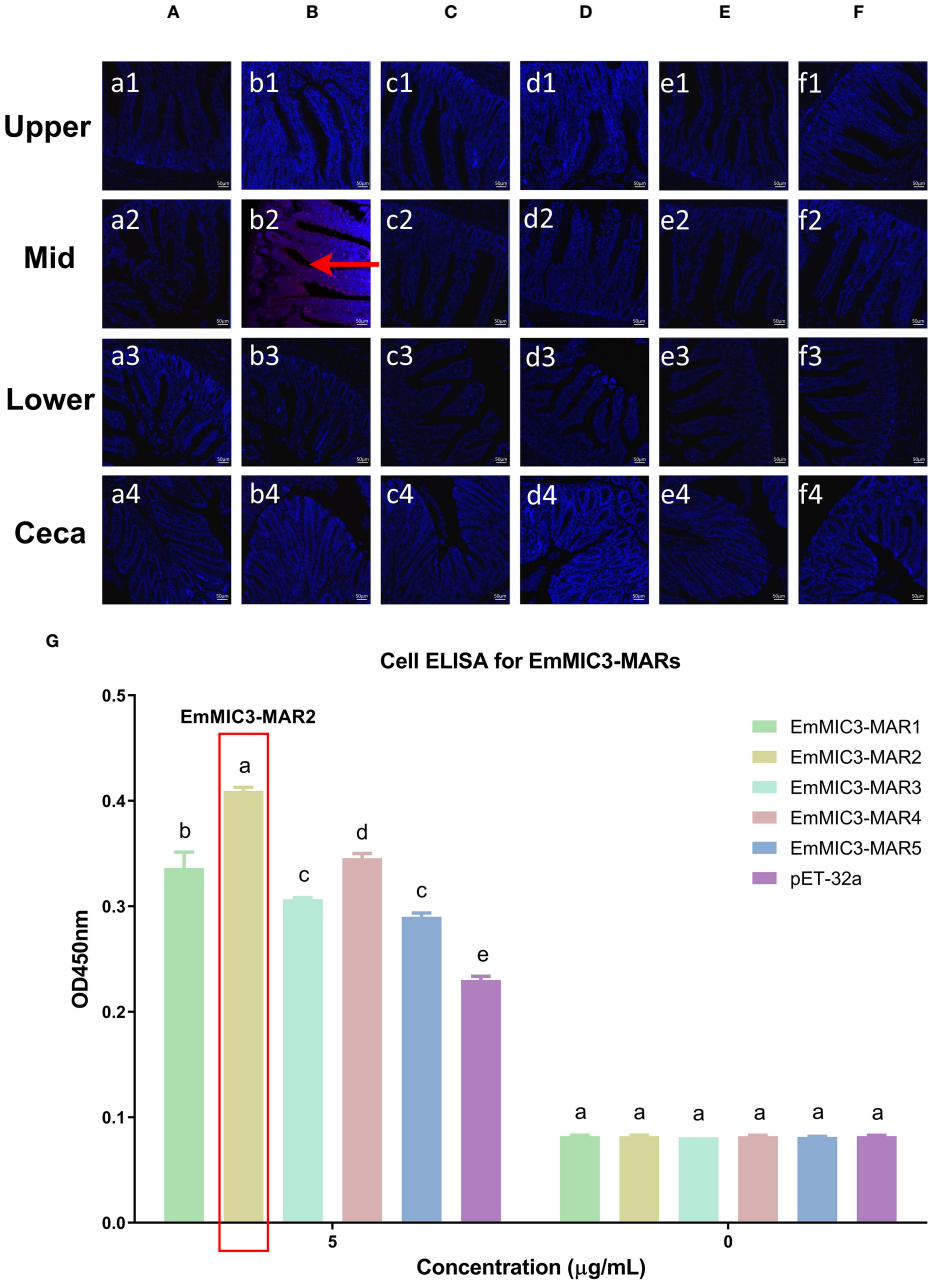
Binding properties of EmMIC3-MARs to different intestine segments. The upper, mid, lower intestine, and caecum tissues were removed from 2-week-old chicken. The substantial red fluorescence from labeling EmMIC3-MAR2 **(B)** was observed in sections of the mid intestines, whilst the EmMIC3-MAR1 **(A)**, EmMIC3-MAR3 **(C)**, EmMIC3-MAR4 **(D)**, EmMIC3-MAR5 **(E)** and pET-32a **(F)**, did not show significant binding to intestinal tissues. Scale bars: 50 μm. The mid intestinal epithelial cells were isolated and cultivated from 2-week-old chickens. The chicken mid intestinal epithelial cells were co-cultured respectively with the recombinant EmMIC3-MARs (0 μg/mL or 5 μg/mL). EmMIC3-MAR2 was most highly active for binding to mid intestinal epithelial cells among all EmMIC3-MARs, of which was concentration-dependent **(G)**. Among different MARs, the significant difference within the group was shown by different letters (*p* < 0.05).

**Figure 5:**
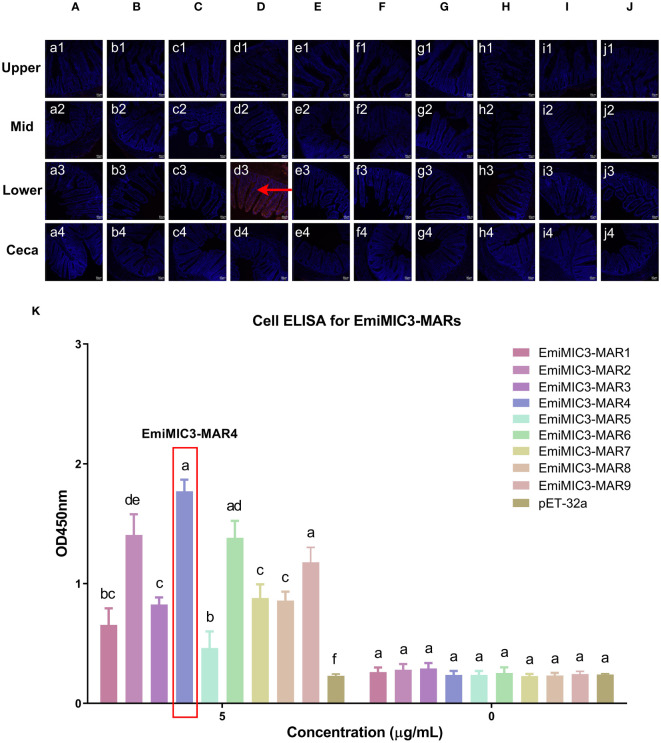
Binding properties of EmiMIC3-MARs to different intestine segments. The upper, mid, lower intestine, and caecum tissues were removed from 2-week-old chickens. EmiMIC3-MAR4 **(D)** showed stronger binding to the lower intestines rather than to other gut tissues (red fluorescence), whilst EmiMIC3-MAR1 **(A)**, EmiMIC3-MAR2 **(B)**, EmiMIC3-MAR3 **(C)**, EmiMIC3-MAR5 **(E)**, EmiMIC3-MAR6 **(F)**, EmiMIC3-MAR7 **(G)**, EmiMIC3-MAR8 **(H)**, EmiMIC3-MAR9 **(I)**, and pET-32a **(J)** did not show significant binding to intestinal tissues. Scale bars: 50μm. The lower intestinal epithelial cells were isolated and cultivated from 2-week-old chickens. The chicken lower intestinal epithelial cells were co-cultured respectively with the recombinant EmiMIC3-MARs (0 μg/mL or 5 μg/mL). EmiMIC3-MAR4 has the strongest binding to lower intestinal epithelial cells **(K)**. Among different MARs, the significant difference within the group was shown by different letters (*p* < 0.05).

To determine more precisely which MAR of EaMIC3, EmMIC3, and EmiMIC3 functions in tissue binding, the binding capability of different EaMIC3-MARs, EmMIC3-MARs, and EmiMIC3-MARs was compared using IFA assays. Among seven EaMIC3-MARs, EaMIC3-MAR3 gave the most robust binding to the upper intestine sections (red fluorescence) (**Figure 3C**, c1), but not to other gut sections, whilst the remaining EaMIC3-MARs and the pET-32a tag protein did not show significant binding to intestinal tissues (**Figures 3A–H**). This indicates that EaMIC3-MAR3 is the dominating domain in the EaMIC3 binding. As for EmMIC3, incubation of gut sections with different EmMIC3-MARs revealed an abundance of EmMIC3-MAR2 staining in sections taken from the mid intestines (**Figure 4B**, b2). Another striking finding was that no red fluorescence was observed from gut sections treated with the pET-32a tag protein, EmMIC3-MAR1, EmMIC3-MAR3, EmMIC3-MAR4, and EmMIC3-MAR5 (**Figures 4A, C–F**), demonstrating the predominant role of EmMIC3-MAR2 in tissue binding of EmMIC3. In addition, a unique binding pattern was presented by EmiMIC3-MAR4, showing strong binding to the lower intestines rather than to other gut tissues (**Figure 5D**, d3). Notably, the other eight EmiMIC3-MARs and the pET-32a tag protein gave no binding to any gut section (**Figures 5A–C, E–J**). This tissue binding pattern of EmiMIC3-MARs suggested that EmiMIC3-MAR4 was a potent domain of EmiMIC3.

### EaMIC3-MAR3, EmMIC3-MAR2, and EmiMIC3-MAR4 exhibited robust interactions with epithelial cells derived from various intestinal tissues

To further validate the tissue staining pattern observed for MARs from EaMIC3, EmMIC3, and EmiMIC3, cell-based ELISA assays were developed and performed using intestine epithelial cells. All of the seven EaMIC3-MARs at varying concentrations could bind to intestinal epithelial cells derived from the upper intestines (**Figure 3I**, **Supplementary File 1**). Compared to the other six EaMIC3-MARs, EaMIC3-MAR3 exhibited the most robust binding signals to epithelial cells in a dose-dependent manner, which is consistent with the IFA results (**Figure 3I**, **Supplementary File 1**). For five EmMIC3-MARs, a sequential two-fold dilution of each EmMIC3-MAR interacted with host epithelial cells isolated from the mid intestines (**Figure 4G**, **Supplementary File 2**). Beyond that, EmMIC3-MAR2 was most highly active for cell binding among all EmMIC3-MARs, which was concentration-dependent (**Figure 4G**, **Supplementary File 2**). Each EmiMIC3-MAR domain could also bind to epithelial cells separated from the lower intestine, and a comparison of the binding capabilities of different EmiMIC3-MARs reveals that EmiMIC3-MAR4 has the strongest binding to cells (**Figure 5K**, **Supplementary File 3**). Therefore, these observations provide more detailed evidence of the roles of EaMIC3-MAR3, EmMIC3-MAR2, and EmiMIC3-MAR4, respectively, for EaMIC3, EmMIC3, and EmiMIC3 binding within the gut of chicken.

To provide a structural basis for the recognition of EaMIC3-MAR3, EmMIC3-MAR2, and EmiMIC3-MAR4, we carried out the three-dimensional structural characterization of these MARs. The three-dimensional structures of the EaMIC3-MAR3, EmMIC3-MAR2, and EmiMIC3-MAR4 are relatively identical, containing α-helix and β-sheet; moreover, the three domains can overlap with each other to a certain extent (**Figures 2D–F**). By characterizing the arrangement of the LxxY and HxS/HxT motifs in three-dimensional space, it was found that the spatial distribution of these two motifs within the EaMIC3-MAR3, EmMIC3-MAR2, and EmiMIC3-MAR4 domains are considerably similar. However, they have slightly different secondary structures; the LxxY motif forms random coils on the EaMIC3-MAR3 (**Figure 2D**), while the α-helix forms on the EmMIC3-MAR2 (**Figure 2E**). The HxS/HxT motif forms a β-sheet on EaMIC3-MAR3, EmMIC3-MAR2, and EmiMIC3-MAR4, respectively (**Figures 2D–F**).

### The antibodies against EaMIC3-MAR3, EmMIC3-MAR2, and EmiMIC3-MAR4 significantly inhibited the invasions of sporozoites into the intestine epithelial cells

**Figure 6:**
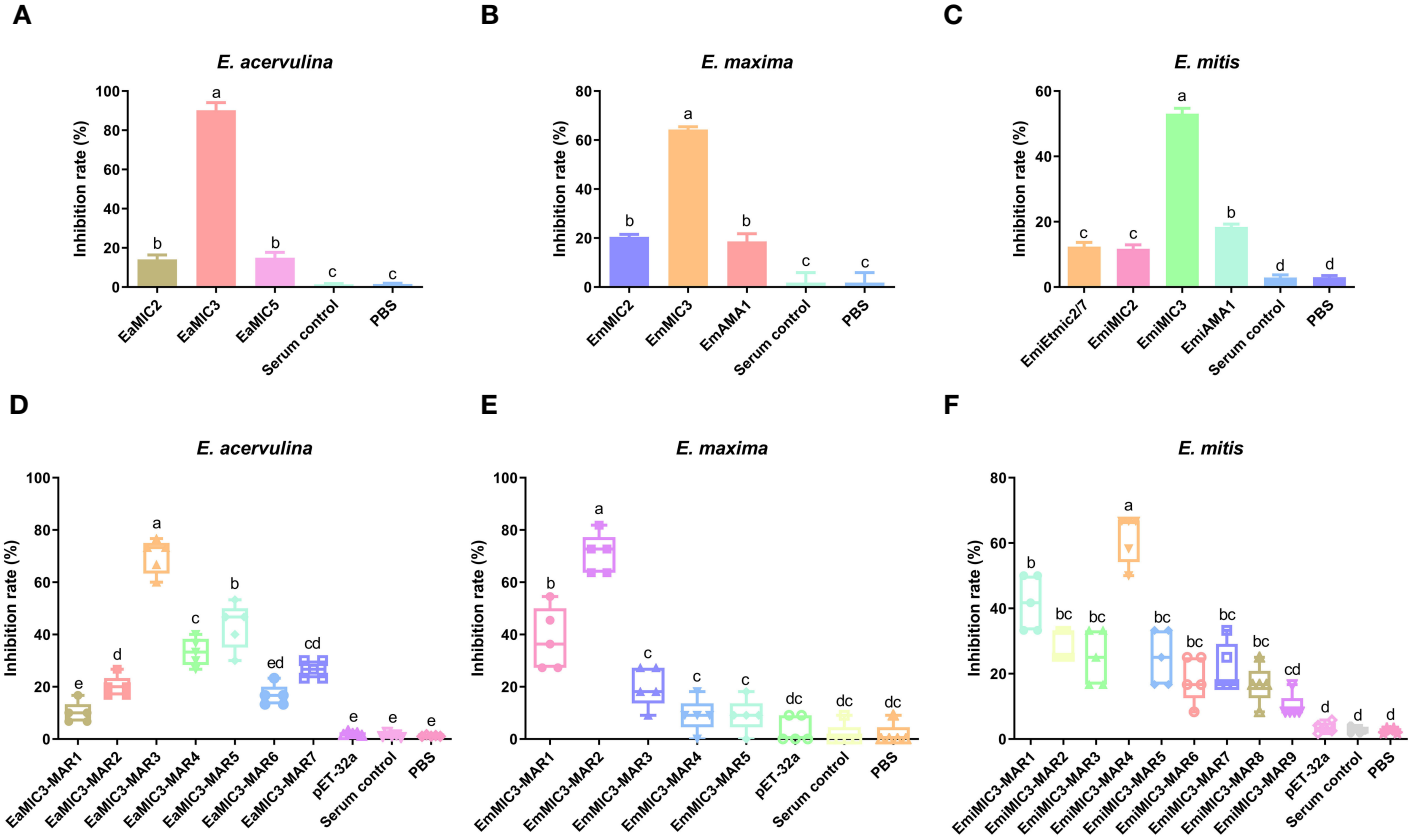
The inhibition effects of sera against MICs and MARs on *Eimeria* sporozoite invasion. *E acervulina* sporozoites were treated with rat anti-EaMIC2, -EaMIC3, -EaMIC5, and -EaMIC3-MARs pAbs, respectively. The sporozoites of *E maxima* were treated with rat anti-EmMIC2, -EmMIC3, -EmAMA1, and -EmMIC3-MARs pAbs, respectively, while *E mitis* sporozoites were blocked with rat anti-EmiMIC3, -EmiMIC2, -EmiEtmic2/7h, -EmiAMA, or -EmiMIC3-MARs pAbs, respectively. The treatment of anti-EaMIC3 pAbs presents the higher inhibition rate than anti-EaMIC2 and anti-EaMIC5 pAb **(A)**. The treatment of anti-EmMIC pAbs exerts much stronger inhibitory activity than anti-EmMIC2 and anti-EmAMA1 pAbs **(B)**. The treatment of anti-EmiMIC3 pAbs was a more potent inhibitor of *E mitis* sporozoite invasion in the lower intestines **(C)**. EaMIC3-MAR3 serum exhibited the highest inhibitory activity in the invasion of *E acervulina* sporozoites **(D)**. Both anti-EmMIC3-MAR1 and anti-EmMIC3-MAR2 pAbs could inhibit *E maxima* sporozoite invasion in cultured mid intestines, and more robust inhibitory activity was observed with EmMIC3-MAR2 antibody compared with EmMIC3-MAR1 antibody in the inhibition experiments **(E)**. The significant inhibition of *E mitis* sporozoite invasion in the lower intestines following the treatment of EmiMIC3-MAR4 antibody **(F)**. The significant difference was shown with different letters (*p* < 0.05).

Given the binding specificity of EaMIC3, EmMIC3, and EmiMIC3, as well as associated MAR domains, we further carried out an inhibition assay using antibodies against different MICs and MARs to assess their roles in sporozoite invasion. As shown in **Figure 6A**, the treatment of anti-EaMIC2, anti-EaMIC3, and anti-EaMIC5 pAbs significantly inhibited the invasion of *E. acervulina* sporozoites to host upper intestines (*P* < 0.05 for each). Moreover, the treatment of anti-EaMIC3 pAbs (90.24%) gave a much higher inhibition rate than anti-EaMIC2 (14.15%) and anti-EaMIC5 pAbs (14.93%) (**Figure 6A**). In addition, *E. maxima* sporozoite invasion of cultured mid intestine tissues could be inhibited by anti-EmMIC2, anti-EmMIC3, and anti-EmAMA1 pAbs (*P* < 0.05), and anti-EmMIC3 pAbs (64.5%) exert much stronger inhibitory activity than anti-EmMIC2 and anti-EmAMA1 pAbs (*P* < 0.05) (**Figure 6B**). Besides, *E. mitis* sporozoites pre-exposed to anti-EmiMIC2, anti-EmiMIC3, ani-EmiEtmic2/7h, and ani-EmiAMA1 pAbs had much lower invading activity compared to the mock control (*P* < 0.05), and anti-EmiMIC3 pAbs was a more potent inhibitor of *E. mitis* sporozoite invasion in the lower intestines (53.09%) (**Figure 6C**).

Likewise, varying anti-MARs antibodies were also prepared to evaluate their inhibitory activity on corresponding MARs during the invasion of sporozoites of three *Eimeria* spp. Of all seven EaMIC3-MARs, EaMIC3-MAR3 serum exhibited the highest inhibitory activity in the invasion of *E. acervulina* sporozoites (*P* < 0.05), and the inhibition rate was 70% (**Figure 6D**). Meanwhile, both anti-EmMIC3-MAR1 and anti-EmMIC3-MAR2 pAbs could inhibit *E. maxima* sporozoite invasion in cultured mid intestines (*P* < 0.05), and more robust inhibitory activity was observed with EmMIC3-MAR2 antibody compared with EmMIC3-MAR1 antibody in the inhibition experiments (70.91%) (*P* < 0.05) (**Figure 6E**). Additionally, of all nine EmiMIC3-MARs, we found significant inhibition of *E. mitis* sporozoite invasion in the lower intestines following the treatment of EmiMIC3-MAR4 antibody (*P* < 0.05), while the other eight EmiMIC3-MAR antibodies did not show any inhibitory activities (**Figure 6F**).

### Binding domain substitutions revealed MAR-mediated site specificity of *Eimeria* invasion

**Figure 7:**
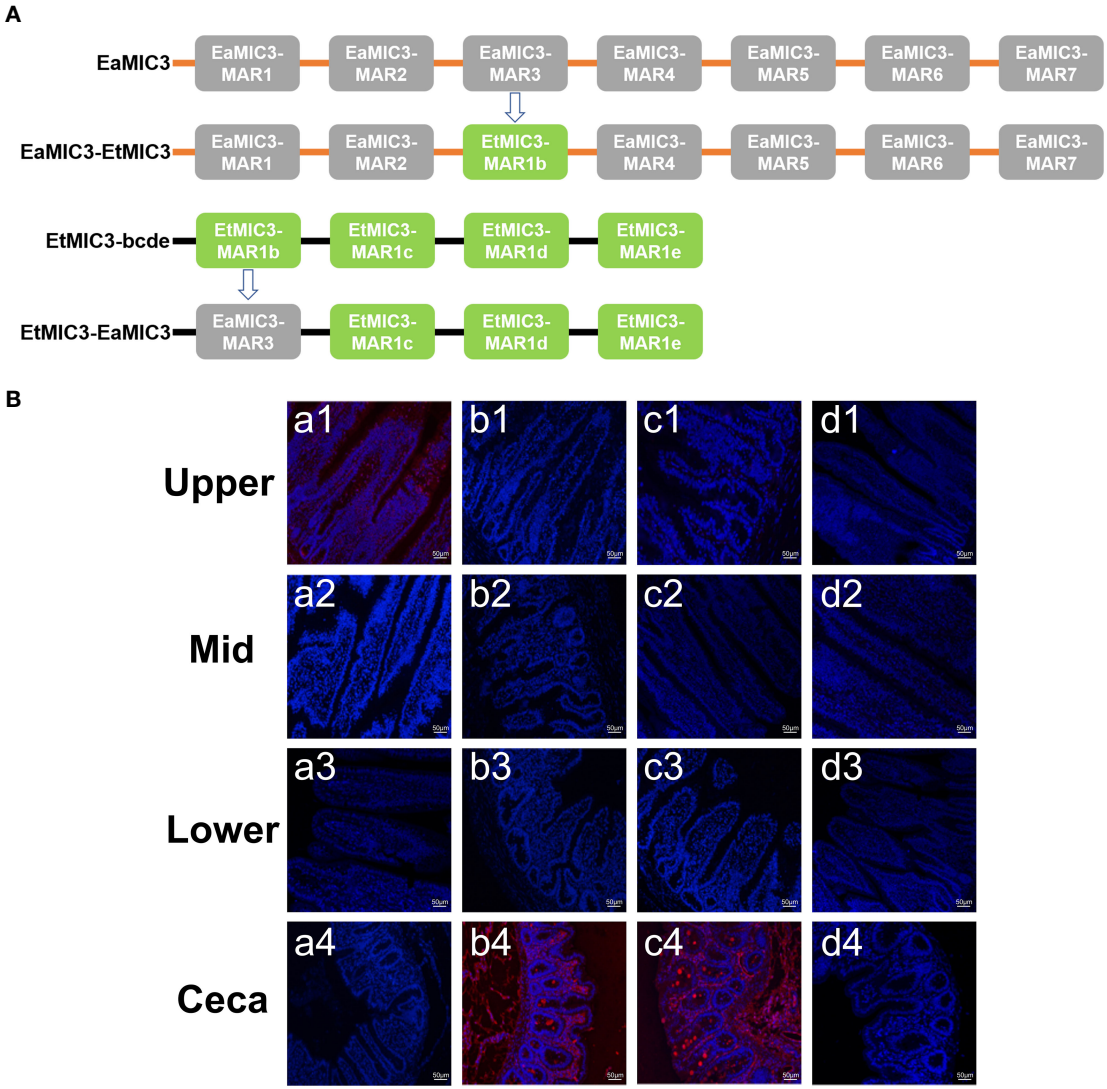
Binding capacity of chimeric proteins to different intestine segments. The structure of EaMIC3-EtMIC3 and EtMIC3-EaMIC3 **(A)**. The upper, mid, lower intestine, and caecum were removed from 2-week-old chicken. The immunohistochemical assay indicated that unlike EtMIC3 with a MAR1b domain which primarily binds to the caecum, EtMIC3-EaMIC3 with a unique EaMIC3-MAR3 domain could solely give a robust binding to the upper intestine **(B)**, a1). Instead of binding to the upper intestine, EaMIC3-EtMIC3 with a particular EtMIC3-EaMIC3 domain can bind to the caecum only **(B)** b4. EtMAR1b can bind to the caecum only **(B)** c4. pET-32a can not bind to any intestinal tissues **(B)**, d1-d4. Scale bars: 50 μm.

To further elucidate the predominant roles of EaMIC3-MAR3, EmMIC3-MAR2, and EmiMIC3-MAR4 in EaMIC3-mediated, EmMIC3-mediated, and EmiMIC3-mediated tissue binding, we selected the EaMIC3-MAR3 domain and EtMIC3-MAR1b domain to construct chimeric EaMIC3-EtMIC3 and EtMIC3-EaMIC3 proteins (**Figure 7A**) for comparison studies. The immunohistochemical assay indicated that, unlike EtMIC3 with a MAR1b domain which primarily binds to the caecum, EtMIC3-EaMIC3 with a unique EaMIC3-MAR3 domain could solely give a robust binding to the upper intestine (**Figure 7B**, a1). Besides, instead of binding to the upper intestine, EaMIC3-EtMIC3 protein with a particular EtMIC3-MAR1b domain can bind to the caecum only (**Figure 7B**, b4). Notably, all these observations suggested that without the core MAR domain, either EaMIC3 or EtMIC3 would lose binding specificity to host tissues. Collectively, the EaMIC3-MAR3 domain indeed contributed to EaMIC3-mediated tissue tropisms for *E. acervuline* within the gut of chicken.

### EmiMIC3-MAR4 elicited partial protection against *Eimeria mitis* infection

To explore whether immunization with EmiMIC3, EmiMIC3-MAR4, and EmiMIC3-MAR5 could induce protection against *E. mitis* infection, we performed active and passive immunization trials in chickens. The protective efficacy of recombinant EmiMIC3, EmiMIC3-MAR4, and EmiMIC3-MAR5 and their pAbs were described in **Tables 1** and **2**, respectively. In the active vaccination trial, compared to the challenge control group, birds immunized with recombinant EmiMIC3, EmiMIC3-MAR4, and EmiMIC3-MAR5 had much-improved body weight gains (*P* < 0.05) (**Table 1**). Following the challenge with *E. mitis* oocysts, there was a statistically significant reduction in oocyst shedding in EmiMIC3-vaccinated, EmiMIC3-MAR4-vaccinated, and EmiMIC3-MAR5-vaccinated groups of birds compared to challenged control groups (*P* < 0.05) (**Table 1**). It was noted that the EmiMIC3 protein conferred substantially higher protection efficacy than EmiMIC3-MAR4 and EmiMIC3-MAR5 (*P* < 0.05) (**Table 1**), and EmiMIC3-MAR4 was more potent in inducing protection against *E. mitis* than EmiMIC3-MAR5 (*P* < 0.05) (**Table 1**). In the passive immunization trial, a similar protection pattern was observed. Immunization with antibodies against EmiMIC3, EmiMIC3-MAR4, and EmiMIC3-MAR5 elicited encouraging levels of protection in *E. mitis*-challenged birds, as instanced by much better body weight gain (*P* < 0.05) and reduced oocyst output (*P* < 0.05) in the feces compared to challenged controls (**Table 2**).

**Table 1:** Protective efficacy of recombinant EmiMIC3, EmiMIC3-MAR4 and EmiMIC3-MAR5 proteins against challenge infection with *E. mitis*.

Groups	Average body weight gains(g)	Oocyst output(lg)	Oocyst decrease ratio (%)
Unchallenged control	84.90 ± 6.25^d^	0.00 ± 0.00^a^	100
pET-32a protein control	19.29 ± 6.74^a^	4.34 ± 1.22^c^	28.14
Recombinant EmiMIC3 protein	74.75 ± 1.71^c^	1.59 ± 0.23^b^	73.67
Recombinant EmiMIC3-MAR4 protein	70.89 ± 4.65^bc^	2.06 ± 0.90^b^	65.89
Recombinant EmiMIC3-MAR5 protein	65.76 ± 5.04^b^	3.47 ± 0.55^b^	42.54
Challenged control	19.11 ± 7.18^a^	6.04 ± 2.26^d^	0.00

The difference (p < 0.05) between groups is shown in each column with different letters.

**Table 2:** Protective efficacy of recombinant EmiMIC3, EmiMIC3-MAR4 and EmiMIC3-MAR5 protein polyclonal antibodies against challenge infection with *E. mitis*.

Groups	Average body weight gains(g)	Oocyst output(lg)	Oocyst decrease ratio (%)
Unchallenged control	67.35 ± 7.16^c^	0.00 ± 0.00^a^	100
pET-32a protein polyclonal antibody control	23.73 ± 4.83^a^	3.97 ± 1.26^b^	3.40
Recombinant EmiMIC3 protein polyclonal antibody	63.84 ± 6.33^bc^	1.04 ± 0.23^a^	74.45
Recombinant EmiMIC3-MAR4 protein polyclonal antibody	53.28 ± 5.96^bc^	1.73 ± 0.55^a^	58.04
Recombinant EmiMIC3-MAR5 protein polyclonal antibody	45.77 ± 5.16^b^	1.74 ± 0.46^a^	57.6
Challenged control	20.81 ± 6.11^a^	4.11 ± 0.78^b^	0.00

The difference (p < 0.05) between groups is shown in each column with different letters.

## Discussion

The distinct host and tissue tropisms of chicken coccidia parasites have been an unresolved debate in poultry studies, with far-reaching implications for understanding the invasion machinery of these parasites and developing novel therapeutics. Unlike *T. gondii* and *N. caninum* which can infect any warm-blooded nucleated cells, the vast majority of *Eimeria* spp., including all of the economically important ones that cause disease in poultry, replicate only within epithelial enterocytes of the different host intestinal tracts, by contrast (9). In recent years, slow but significant progress has been made to reveal the molecular basis for the site specificity of *Eimeria* parasites, and various molecules contributing to cell recognition and invasion, such as MICs, have been identified. Still, research on how *Eimeria* parasites build their invasion machinery lags far behind that of *Toxoplasma*, in addition to *Plasmodium*. Of all seven chicken coccidia parasites, the invasion machinery of *E. tenella* was best studied, and detailed characterization of EtMIC repertoire was carried out from structural and functional aspects, mainly for EtMIC3. However, limited investigations on MICs of other chicken *Eimeria* parasites have hindered the elucidation of their specific molecular interactions with hosts and the characterization of their roles at the host-parasite interface. Thus, it has prompted our group to further study EaMICs, EmMICs, and EmiMICs to find rationalization of differing host and tissue tropisms for *E. acervuline*, *E. maxima*, and *E. mitis*. We compared binding profiles of varying EaMICs, EmMICs, and EmiMICs to host intestines and determined the key MAR domain from EaMIC3, EmMIC3, and EmiMIC3 in MIC biology to demonstrate the molecular basis for host tissue tropisms of *E. acervuline*, *E. maxima*, and *E. mitis*.

MICs released from the secretory organelles mediate the initial specific high-affinity interaction between host cells and apicomplexan parasites and play an important role in host cell recognition and attachment. They are critically involved in the invasion process of apicomplexan parasites, allowing parasites to target a diverse range of cell surface oligosaccharide epitopes. Therefore, the host specificity and tissue tropism of apicomplexan parasites appear to be associated with the repertoire and specificity of the MICs that they produce. A family of MICs has been characterized in *E. acervuline*, *E. maxima*, and *E. mitis*, respectively, including seven EaMICs, six EmMICs, and five EmiMICs. Given that *E. acervuline*, *E. maxima*, and *E. mitis* exert distinct tissue tropisms (*E. acervuline* invades the upper intestine; *E. maxima* develops within the mid intestine; *E. mitis* parasitizes the lower intestine) and EaMICs, EmMICs, and EmiMICs are likely to be major determinants for this; therefore, we determined the binding specificity of EaMICs, EmMICs, and EmiMICs to different gut segments in this study. The immunohistochemical assay revealed a restricted tissue staining pattern: of all EaMICs, only EaMIC3 could bind to sections of the upper intestine; EmMIC3 could solely bind to the mid intestine, whereas other EmMICs showed no binding to any intestinal tissues; EmiMIC3 was the only EmiMIC that could bind to the lower intestine. All the tissue binding profiles suggested that EaMIC3, EmMIC3, and EmiMIC3, respectively, may play a key role in directing *E. acervuline*, *E. maxima*, and *E. mitis* to their specific parasitism location. Taking that the deployment of EtMIC3 governs host cell recognition and attachment into account, we speculated that all the MIC3s of *Eimeria* play a dominant role in determining the site specificity of different *Eimeria* parasites during the invasion. However, it warrants further investigation.

MICs are characterized by their composition of a modular arrangement of adhesive domains, such as the Apple/PAN motif, epidermal growth factor (EGF-like) domain, Galectin-like domain, Type I like repeats (TSR-like) domain, and MAR domain, which were responsible for the entry of parasites into host cells (9). Of note, the MAR domain that governs interaction with host ligands has been shown to be restricted to coccidial parasites. As a result, for *Eimeria* spp., sequence and structural variations of MAR domains lead to variant capacities for sialyl ligand binding, which account for the divergence in host and site specificities of apicomplexan parasites. There are seven MAR domains for EaMIC3, five MAR domains for EmMIC3, and nine MAR domains for EmiMIC3. Multiple sequence alignment revealed that each of EaMIC3-MARs, EmMIC3-MARs, and EmiMIC3-MARs contains seven cysteine residues, with the exception of an incomplete repeating sequence. These cysteine residues might play a role in forming the disulfide bond in *E. acervulina*, *E. maxima*, and *E. mitis*, respectively. The presence of multiple disulfide bonds stabilizes the core structure and enhances the stability of the protein molecule against external influences. By conducting a comparative analysis of the amino acid sequences of the MAR domains of three *Eimeria* species, we discovered that each complete MAR domain possesses a relatively conserved type I MAR structure, characterized by the presence of the HxT/HxS and LxxY motifs. The LxxY motif, which takes the form of an α1 helix/loop structure, plays a crucial role in facilitating interactions between the MAR domain and sialyl saccharides, while simultaneously preventing contact with N-hydroxylacetyl sialic acid. As a result, this motif contributes significantly to the specificity of the EaMIC3-MAR, EmMIC3-MAR, and EmiMIC3-MAR domains in their binding. The HxT/HxS motif, on the other hand, operates through hydrogen bonding and π-stacking with conserved histidine and threonine residues, modulating binding to sialic acid polysaccharides. This recognition pattern of sialic acids is similar to that observed in other pathogen lectins, suggesting that the HxT/HxS motif also contributes to the binding properties of EaMIC3-MARs, EmMIC3-MARs, and EmiMIC-MARs. In this study, we further compared the binding capabilities of these EaMIC3-MARs, EmMIC3-MARs, and EmiMIC3-MARs to host intestinal tissues. Of all EaMIC3-MARs, EaMIC3-MAR3 showed significant binding to the upper intestines, and EmMIC3-MAR2 gave the most robust binding to the mid intestines among the five EmMIC3-MARs. Besides, EmiMIC3-MAR4 had a more robust binding to the lower intestines than other EmiMIC3-MARs. All these binding patterns suggested the predominant roles of EaMIC3-MAR3, EmMIC3-MAR2, and EmiMIC3-MAR4 in the tissue binding mediated by EaMIC3, EmMIC3-MAR2, and EmiMIC3, respectively. With the aim to confirm this, we next carried out cell-based binding ELISA assays. The results showed that all EaMIC3-MARs, EmMIC3-MARs, or EmiMIC3-MARs could interact with intestinal epithelial cells, but the binding capacity of each MAR domain varied. Consistent with the observation from the IFA assays, among these different MAR domains, EaMIC3-MAR3 of *E. acervulina*, EmMIC3-MAR2 of *E. maxima*, and EmiMIC3-MAR4 of *E. mitis* also exerted the highest binding capacity.

In this study, to further understand the restricted binding properties of EaMIC3-MAR3, EmMIC3-MAR2, and EmiMIC3-MAR4, chimeric EaMIC3-EtMIC3 or EtMIC3-EaMIC3 proteins were constructed by substituting the EtMIC3-MAR1b or EaMIC3-MAR3 domains in EaMIC3 or EtMIC3, respectively. Results from the intestinal tissue binding experiment with the chimeric proteins showed that EtMIC3-EaMIC3 with an EaMIC3-MAR3 domain was able to bind to the upper intestine of a chicken but not to the cecum. Conversely, EaMIC3-EtMIC3 with an EtMIC3-MAR1b domain exhibited robust binding to the cecum but lost its ability to interact with the host’s upper intestine. These findings demonstrate the crucial role of the MAR domains in governing interaction with host cells and indicate that, in *E. acervuline*, the specificity of the EaMIC3 function is largely determined by the EaMIC3-MAR3 domain. In addition to domain substitution tests, we also evaluated the efficacy of MIC and MAR antibodies in blocking sporozoite invasion mediated by MICs or MARs. Our findings showed that *E. acervulina* sporozoites that invade the upper intestine of chickens could be effectively blocked with EaMIC3 antiserum. While EaMIC2 and EaMIC5 antisera also exhibit some inhibitory effects, they are not as potent as EaMIC3. The pathogenicity of *E. maxima* sporozoites was found to be effectively inhibited by EmMIC3 antiserum when compared with EmMIC2 and EmAMA1 antisera. Similarly, EmiMIC3 antiserum showed promising efficacy in restricting the invasion of *E. mitis* sporozoites to the lower intestine compared to EmiMIC2, EmiEtmic2/7h, and EmiAMA1 antisera. A similar trend was observed with the use of anti-MAR antibodies, with EaMIC3-MAR3, EmMIC3-MAR2, and EmiMIC3-MAR4 antisera exhibiting the highest inhibitory effects. In particular, rat anti-EmMIC3-MAR2 was found to be the most effective in blocking the invasion of *E. maxima* sporozoites, thus indicating that EmMIC3-MAR2 may play a crucial role in the entire invasion process. The ability of *E. acervuline* or *E. mitis* sporozoites to invade was significantly impaired when they were treated with rat anti-EaMIC3-MAR3 or anti-EmiMIC3-MAR4 antibodies, making EaMIC3-MAR3 or EmiMIC3-MAR4 the most active of the seven EaMIC3-MARs or nine EmiMIC3-MARs.

Animal experiments have demonstrated the significant role of MICs and MARs in conferring immunity. The EaMIC3 protein was found to be present in the sporozoite stage and located at the apical tip of the sporozoite. Immunization with recombinant EaMIC3 was shown to stimulate strong Th1 immune responses against *E. acervuline* infection and significantly improve weight gain while reducing mortality (25). Similarly, vaccination with recombinant EmiMIC3 protein resulted in reduced weight loss and decreased oocyst output in chickens infected with *E. mitis* (32). The use of *Salmonella typhimurium* X4550 carrying two EtMIC3-C-MAR domains as a live attenuated vaccine showed promising results in terms of improving immunogenic protection in the host (39). The current study investigated the protective efficacies of the EmiMIC3, EmiMIC3-MAR4, and EmiMIC3-MAR5 proteins and their polyclonal antibodies. The results indicated that chickens immunized with EmiMIC3-MAR5 and EmiMIC3-MAR4 elicited comparatively better protection. The protective effects of EaMIC3-MAR3 and EaMIC3-MAR6 from *E. acervulina* were also demonstrated, with EaMIC3-MAR3 exhibiting the best binding capacity to the upper section of the intestine and providing improved immunoprotective efficacy (40).

To conclude, our findings suggested that EaMIC3, EmMIC3, and EmiMIC3 play crucial roles in the site specificity of *E. acervulina*, *E. maxima*, and *E. mitis*, respectively, and EaMIC3-MAR3, EmMIC3-MAR2, and EmiMIC3-MAR4 are the underlying factors behind their variable tissue tropisms. The results provided valuable insights into the modes of action of *Eimeria* MICs on a molecular basis, thus offering a rational explanation for the distinct tissue tropisms observed across *E. acervuline*, *E. maxima*, and *E. mitis*.

## Materials and methods

### Ethics statement

Animal experiments were conducted in strict accordance with the guidelines of the Animal Welfare and Ethical Review Board of Nanjing Agricultural University, China. All animal studies and protocols were evaluated and approved by the Institutional Animal Care and Use Committee of Nanjing Agricultural University (Approval number: 2012CB120750).

### Parasites and animals

*E. acervulina* Nanjing strain, *E. maxima* Nanjing strain, and *E. mitis* Nanjing strain were obtained and propagated in conditions as previously described (25, 30, 32). New-born Hy-Line layer chickens were raised under coccidia-free conditions. The chickens were provided with anticoccidial drug-free feed and water. SD rats of 30 days old were purchased from the Comparative Medicine Center of Yangzhou University (Yangzhou, China) and kept in specific-pathogen-free conditions for antiserum collection.

### Sequence alignment and three-dimensional structure analysis of MIC3-MARs of *Eimeria acervulina*, *Eimeria maxima*, and *Eimeria mitis*

The full sequence of the *EmMIC3* gene was obtained using the rapid-amplification of cDNA ends (RACE) method with the forward gene-specific primers (**Table S1**) as previously described (24). The complete open reading frame (ORF) of EmMIC3 was submitted to GenBank under Accession No. KU93609. All the MARs identified in EaMIC3 (AMN15064.1), EmMIC3 (AOY42085.1), and EmiMIC3 (AXC32915.1) were analyzed using NCBI (https://www.ncbi.nlm.nih.gov/nuccore). Clustal X and ESPript 3 (http://espript.ibcp.fr/ESPript/ESPript/) were used to align the sequences of MARs of *E. acervulina*, *E. maxima*, and *E. mitis*. The pyMOL software (Version 2.5.4) was employed to construct the three-dimensional structures of the MAR3 of EaMIC3 (EaMIC3-MAR3), MAR2 of EmMIC3 (EmMIC3-MAR2) and MAR4 of EmiMIC3 (EmiMIC3-MAR4).

### Productions of recombinant proteins of MICs and MARs

The ORFs of *EaMIC2*, *EaMIC3*, *EaMIC5*, *EmMIC2*, *EmMIC3*, *EmAMA1*, *EmiMIC2*, *EmiMIC3*, *EmiAMA1*, and *EmiEtmic-2/7* genes were amplified by PCR using specific primers designed according to available mRNA sequences of EaMIC2 (KR063282), EaMIC3 (KU359773), EaMIC5 (KF922373), EmMIC2 (FR718971.1), EmMIC3 (KU93609), EmAMA1 (FN813221.1), EmiMIC2 (XM013494084.1), EmiMIC3 (MG888670.1), EmiAMA1 (XM013496458.1) and EmiEtmic-2/7 (CDJ36057.1). The primers are listed in **Table S2**. Corresponding MAR domains of EaMIC3 (EaMIC3-MARs), EmMIC3 (EmMIC3-MARs), and EmiMIC3 (EmiMIC3-MARs) were then amplified by PCR using specific primers (**Table S3**). The ORFs of EaMIC2, EaMIC3, EaMIC5, EmMIC2, EmMIC3, EmAMA1, EmiMIC2, EmiMIC3, EmiAMA, EmiEtmic-2/7, EaMIC3-MARs, EmMIC3-MARs, EmiMIC3-MARs and were each ligated into the pET-32a plasmid (Sigma-Aldrich, St. Louis, MO) and expressed in *Escherichia coli* BL21 (DE3) (Sigma-Aldrich). Subsequently, the expressed recombinant proteins were purified by His-Trap columns (GE Healthcare, Piscataway, NJ) using previously described strategies (29). Purified proteins were then stored at -80°C until further analysis. Meanwhile, the fusion proteins of pET-32a used as mock control were also prepared with the same procedure.

### Productions of chimeric proteins EaMIC3-EtMIC3 and EtMIC3-EaMIC3

To specify tissue tropism characteristics of *E. acervulina*, two chimeric proteins, namely EaMIC3-EtMIC3 and EtMIC3-EaMIC3, were generated. EaMIC3-EtMIC3 protein was produced by the replacement of EaMIC3-MAR3 fragments with the EtMIC3-MAR1b domain. As a result, EaMIC3-EtMIC3 comprised sequences encompassing EaMIC3-MAR1, EaMIC3-MAR2, EtMIC3-MAR1b, EaMIC3-MAR4, EaMIC3-MAR5, EaMIC3-MAR6, and EaMIC3-MAR7. Likewise, the chimeric EtMIC3-EaMIC3 was constructed by replacing the EtMIC3-MAR1b domain with EaMIC3-MAR3 residues; therefore, it consisted of the sequences of EaMIC3-MAR3, EtMIC3-MAR1c, EtMIC3-MAR1d, and EtMIC3-MAR1e. Specific primers used to construct the chimeric MICs are listed in **Table S4**. Chimeric EaMIC3-EtMIC3 and EtMIC3-EaMIC3 constructs were cloned into the pET-32a expression vector, and recombinant EaMIC3-EtMIC3 and EtMIC3-EaMIC3 proteins were then obtained as stated above.

### Generations of polyclonal antibodies against recombinant proteins

Rat pAbs against rEaMIC2, rEaMIC3, rEaMIC5, rEmMIC2, rEmMIC3, rEmAMA1, rEmiMIC2, rEmiMIC3, rEmiAMA1, rEmiEtmic-2/7, rEaMIC3-MARs, rEmMIC3-MARs, rEmiMIC3-MARs, rEtMIC3-MARs, and pET-32a tag protein were each prepared as previously described (41). Briefly, purified recombinant proteins (200 μg) were emulsified with complete Freund’s adjuvant (Sigma-Aldrich) thoroughly, and the first injection was administered to SD rats. Two weeks later, proteins (200 μg) emulsified with incomplete Freund’s adjuvant (Sigma-Aldrich) were given as the second immunization. Later on, the third and fourth administrations were given at a 7-day interval as above. The rats were bled for serum collection seven days after the last injection. Small aliquots of antiserum were stored at -20°C for further usage.

### Binding profiles of MICs and MARs to small intestine and caecum

The binding abilities of recombinant MICs and MARs to the upper, mid, and lower intestine and caecum were evaluated by the immunofluorescence assay (IFA). Varying small intestine samples (3 cm in length), including upper intestine (5 cm from the stomach), mid intestine (revolving around yolk stalk), and lower intestine (5 cm from recta), as well as caecum tissues, were removed immediately from 2-week-old Hy-Line layer chickens post-mortem. They were fixed in 4% paraformaldehyde and subsequently subjected to dehydration, paraffin embedding, and sectioning (10 μm). To minimize non-specific staining, sections were blocked overnight with 5% bovine serum albumin (BSA, Sigma-Aldrich) in tris-buffered saline with Tween 20 (TBST). The recombinant proteins of MICs and MARs (5μg/mL for each) were incubated with the sections of the upper, mid, and lower intestines and caecum for 30 min at room temperature, respectively. Sections were washed 3 times with TBST and then incubated with rat sera against MICs and MARs of *E. acervulina*, *E. maxima*, and *E. mitis* (1:100 in dilution) for 1 h, respectively. After washing 3 times, sections were incubated with goat anti-rat Alexa Fluor 568 second antibody (red) and later incubated with 4,6-diamidino-2-phenylin-dole (DAPI; blue). After three washes, the slides were mounted with coverslips and analyzed under a laser confocal microscope (Zeiss LSM 710, Germany). The comparison of the binding capability to the upper, mid, and lower small intestines was carried out among different MICs of *E. acervulina* (EaMIC2, EaMIC5, and EaMIC3), *E. maxima* (EmMIC3, EmMIC2, and EmAMA1), and *E. mitis* (EmiMIC3, EmiMIC2, EmiAMA1, and EmiEtmic-2/7), respectively. Meanwhile, the differences in the binding capability of seven MARs of *E. acervulina*, five MARs of *E. maxima*, and nine MARs of *E. mitis* were also investigated.

### Cell binding assay

Cell ELISA was carried out to determine the interaction between MARs and host intestinal epithelial cells. The samples (10 cm in length) of the upper, mid, and lower intestines were collected from 2-week-old chickens. The isolation and cultivation of intestinal epithelial cells of chickens were conducted using a standard procedure as described elsewhere (42, 43). Purified intestinal epithelial cells were seeded in 96-well high-binding culture plates and then incubated with recombinant MARs (0 μg/ml to 100 μg/ml) for 1 h at 41°C with 5% CO_2_. After the centrifuge, cell culture fluid was discarded, and the cells were washed three times with phosphate buffer saline (PBS). Cells were fixed in 4% paraformaldehyde for 10 min at room temperature. After three washes in TBST, cells were blocked with 4% BSA in TBST for 1 h. Subsequently, cells were incubated with rat anti-EaMIC3, -EmMIC3, or -EmiMIC3 serum (1:500 in dilution) for 1 h, respectively. After three washes with TBST, cells were incubated with Horseradish Peroxidase (HRP)-conjugated goat anti-rat IgG (Bioworld, Nanjing, Jiangsu, China) for 1 h. After washing, 3,3’,5,5’-Tetramethylbenzidine (TMB; Sigma-Aldrich) were added in the dark at room temperature for 5min. The reaction was stopped with 2 mol/L H_2_SO_4,_ and the values were measured at 450nm (OD_450_) with an automated ELISA reader (Thermo Multiskan™ FC, USA).

### Binding profiles of chimeric proteins to small intestine and caecum

The effects of MAR substitutions on the abilities of chimeric EaMIC3-EtMIC3 and EtMIC3-EaMIC3 proteins to bind to the intestine were also analyzed by IFA assays as above. Recombinant proteins of EaMIC3-EtMIC3, EtMIC3-EaMIC3, and EtMIC3-MAR1b were incubated with the sections of the upper, mid, and lower intestines and caecum, respectively. For EtMIC3-EaMIC3 and EtMIC3-MAR1b, rat anti-EtMIC3 (1:100 in dilution) was used for the determination of binding, whereas for EaMIC3-EtMIC3, rat anti-EaMIC3 (1:100 in dilution) was used for IFA assays.

### Inhibition experiments

The effects of antiserum against MICs and MARs on the invasion of sporozoites into the intestinal epithelial cells were determined using an *in vitro* inhibition assay. The collection and purification of sporozoites were carried out as previously described (44). Purified sporozoites were blocked by varying pAbs with a final antibody titer of 2^10^ in 10mL PBS for 30 min at 4°C by gently rotating. For *E. acervulina* sporozoites, they were treated with rat anti-EaMIC2, -EaMIC3, -EaMIC5, and -EaMIC3-MARs pAbs, respectively. The sporozoites of *E. maxima* were treated with rat anti-EmMIC2, -EmMIC3, -EmAMA1, and -EmMIC3-MARs pAbs, respectively, while *E. mitis* sporozoites were blocked with rat anti-EmiMIC3, -EmiMIC2, -EmiEtmic2/7h, -EmiAMA1 or -EmiMIC3-MARs pAbs, respectively. Sporozoites blocked with control rat serum and PBS were set as controls. After three washes with PBS, sporozoites were resuspended in 2mL PBS. The upper, mid, and lower intestine segments (5 cm in length) were taken immediately from 2-week-old Hy-Line layer chickens post-mortem and soaked in preheated Hanks Balanced Salt Solution (HBSS; Gibco, Grand Island, NY) (41°C) with one end of each intestine segment ligatured. Pretreated sporozoites (1 × 10^7^) of *E. acervulina*, *E. maxima*, and *E. mitis* in 2mL PBS were poured into the upper, mid, and lower intestine segments, respectively. After ligating another end of intestine segments, intestine samples were incubated in PBS with gentle shaking at 41°C for 15 min. The sporozoites within the intestinal lumen (not entered into the intestinal epithelial cells) were collected after PBS washing three times. The number of sporozoites collected from the intestine lumen was counted by McMaster’s method (45).

### Immunoprotective effect of the MIC3 and MARs vaccines of *Eimeria mitis* in chickens

The protective roles of EmiMIC3, EmiMIC3-MAR4 (the strongest binding capacity with lower intestine), and EmiMIC3-MAR5 (the weakest binding capacity with lower intestine) against *E. mitis* were evaluated using challenge infection models. Two-week-old coccidia-free chickens with similar weight were randomly divided into six groups (20 birds/group), as shown in **Table 1**. The chickens were immunized intramuscularly with 200 μg of rEmiMIC3, rEmiMIC3-MAR4, or rEmiMIC3-MAR5 proteins. Control birds were immunized with an equal volume of PBS or pET-32a tagged protein. One week after primary immunization, the chickens were given a booster immunization. Seven days after the booster immunization, chickens were orally challenged with 1×10^5^ *E. mitis* oocysts per bird except for the unchallenged chicken. Seven days post-challenge, all the chickens were euthanized for the collection of intestine samples.

For passive immunization trials, twenty-eight-day-old coccidia-free chickens with similar weight were randomly divided into six groups (20/group), as presented in **Table 2**. The birds were orally challenged with 1×10^5^ *E. mitis* oocysts, while unchallenged chickens were inoculated with sterile PBS. The chickens were immunized intravenously with anti-EmiMIC3, anti-EmiMIC3-MAR4, or anti-EmiMIC3-MAR5 pAbs for up to seven days. Each bird was injected with 0.5 mL of antiserum. All the chickens were euthanized by cervical dislocation on day 8 of immunization.

The immunoprotective roles of EaMIC3, EaMIC3-MAR3, and EaMIC3-MAR6 of *E. acervulina*, and EmMIC3, EmMIC3-MAR2, and EmMIC3-MAR5 of *E. maxima* were evaluated by the previous research in our lab (40, 46).

### Statistics analysis

GraphPad Prism 6.0 (GraphPad Software, USA) and SPSS 19 (SPSS Software, IBM, USA) were used to calculate the *p*-Values. The comparison was performed using the one-way analysis of variance (ANOVA) corrected by Dunnett’s tests. The expression levels were denoted as mean ± standard deviation (SD).

## Data availability statement

The original contributions presented in the study are included in the article/**Supplementary Material**. Further inquiries can be directed to the corresponding author.

## Ethics statement

The animal study was approved by Animal experiments were conducted in strict accordance with the guidelines of the Animal Welfare and Ethical Review Board of Nanjing Agricultural University, China. All animal studies and protocols were evaluated and approved by the Institutional Animal Care and Use Committee of Nanjing Agricultural University (Approval number: 2012CB120750). The study was conducted in accordance with the local legislation and institutional requirements.

## Author contributions

YZ: Conceptualization, Data curation, Investigation, Writing – original draft. ML: Conceptualization, Writing – review & editing. ZZ: Investigation, Writing – review & editing. XH: Investigation, Writing – review & editing. JWH: Investigation, Writing – review & editing. JL: Investigation, Writing – review & editing. JMH: Software, Writing – review & editing. XS: Conceptualization, Writing – review & editing. LX: Conceptualization, Writing – review & editing. RY: Conceptualization, Writing – review & editing. XL: Conceptualization, Funding acquisition, Writing – review & editing.

## Supplementary material

Supplementary File 1 Binding of EaMIC3-MARs to fixed upper intestinal epithelial cells determined by ELISA. The upper intestinal epithelial cells were isolated and cultivated from 2-week-old chickens. The chicken upper epithelial cells were co-cultured respectively with the recombinant EaMIC3-MARs. The final concentration of every protein were 12.5 μg/mL **(A)**, 25 μg/mL **(B)**, 50 μg/mL **(C)**, and 100 μg/mL **(D)**. Among different MARs, the significant difference within the group was shown by different letters (*p* < 0.05).

Supplementary File 2 Binding of EmMIC3-MARs to fixed mid intestinal epithelial cells determined by ELISA. The mid intestinal epithelial cells were isolated and cultivated from 2-week-old chickens. The chicken mid intestinal epithelial cells were co-cultured respectively with the recombinant EmMIC3-MARs. The final concentration of every protein were 12.5 μg/mL **(A)**, 25 μg/mL **(B)**, 50 μg/mL **(C)**, and 100 μg/mL **(D)**. Among different MARs, the significant difference within the group was shown by different letters (*p* < 0.05).

Supplementary File 3 Binding of EmiMIC3-MARs to fixed lower intestinal epithelial cells determined by ELISA. The lower intestinal epithelial cells were isolated and cultivated from 2-week-old chickens. The chicken lower intestinal epithelial cells were co-cultured respectively with the recombinant EmiMIC3-MARs. The final concentration of every protein were 12.5 μg/mL **(A)**, 25 μg/mL **(B)**, 50 μg/mL **(C)**, and 100 μg/mL **(D)**. Among different MARs, the significant difference within the group was shown by different letters (*p* < 0.05).
