# Supplementary figures and images for: The microneme adhesive repeat domain of MIC3 protein determined the site specificity of Eimeria acervulina, Eimeria maxima, and Eimeria mitis
Source: Front Immunol. 2023 Nov 8;14:1291379. doi: 10.3389/fimmu.2023.1291379 (PMC10663340; doi:10.3389/fimmu.2023.1291379)

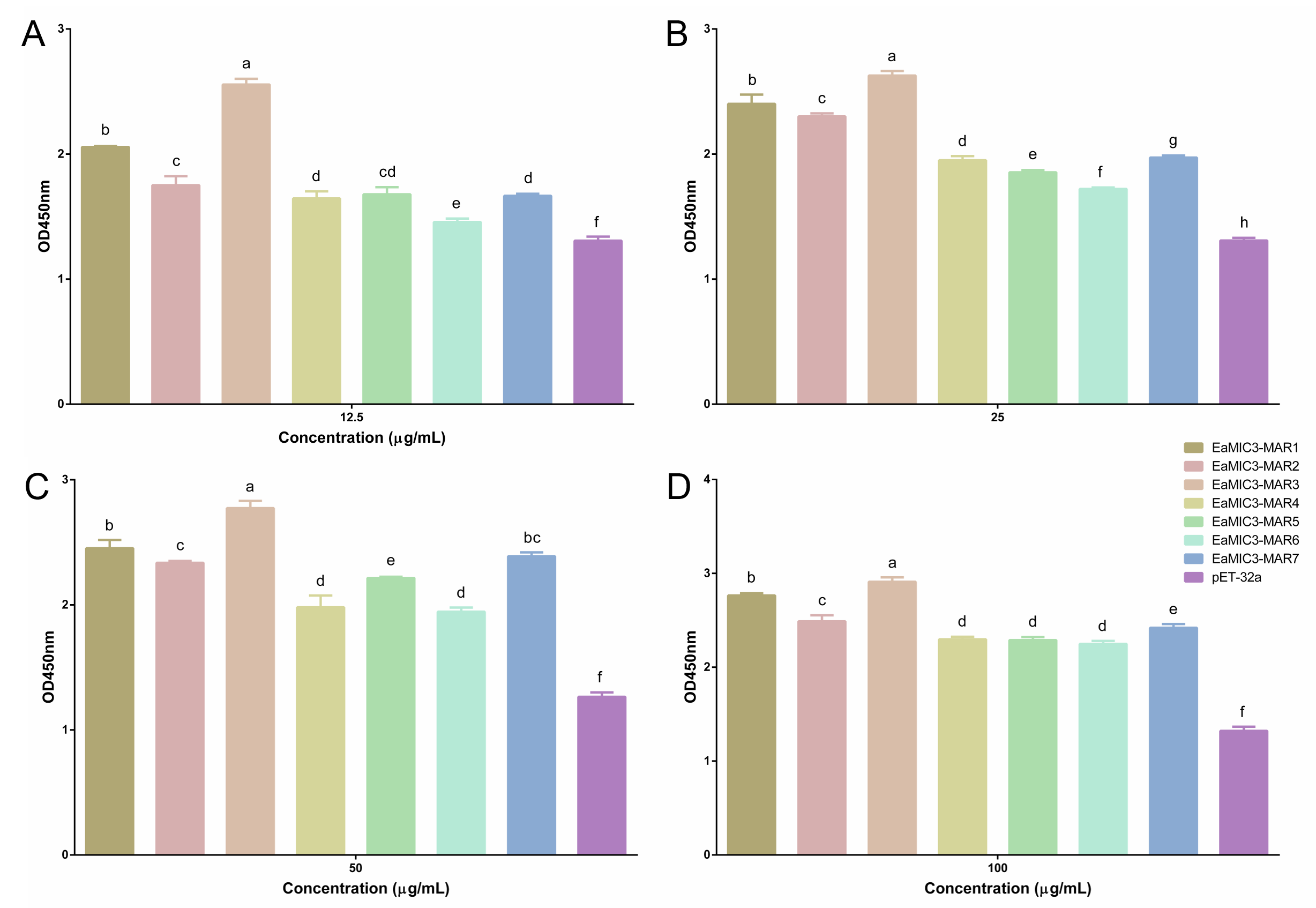

Supplement: Supplementary File 1 — Binding of EaMIC3-MARs to fixed upper intestinal epithelial cells determined by ELISA. The upper intestinal epithelial cells were isolated and cultivated from 2-week-old chickens. The chicken upper epithelial cells were co-cultured respectively with the recombinant EaMIC3-MARs. The final concentration of every protein were 12.5 μg/mL (A), 25 μg/mL (B), 50 μg/mL (C), and 100 μg/mL (D). Among different MARs, the significant difference within the group was shown by different letters (p < 0.05). [file Image_1.tif]

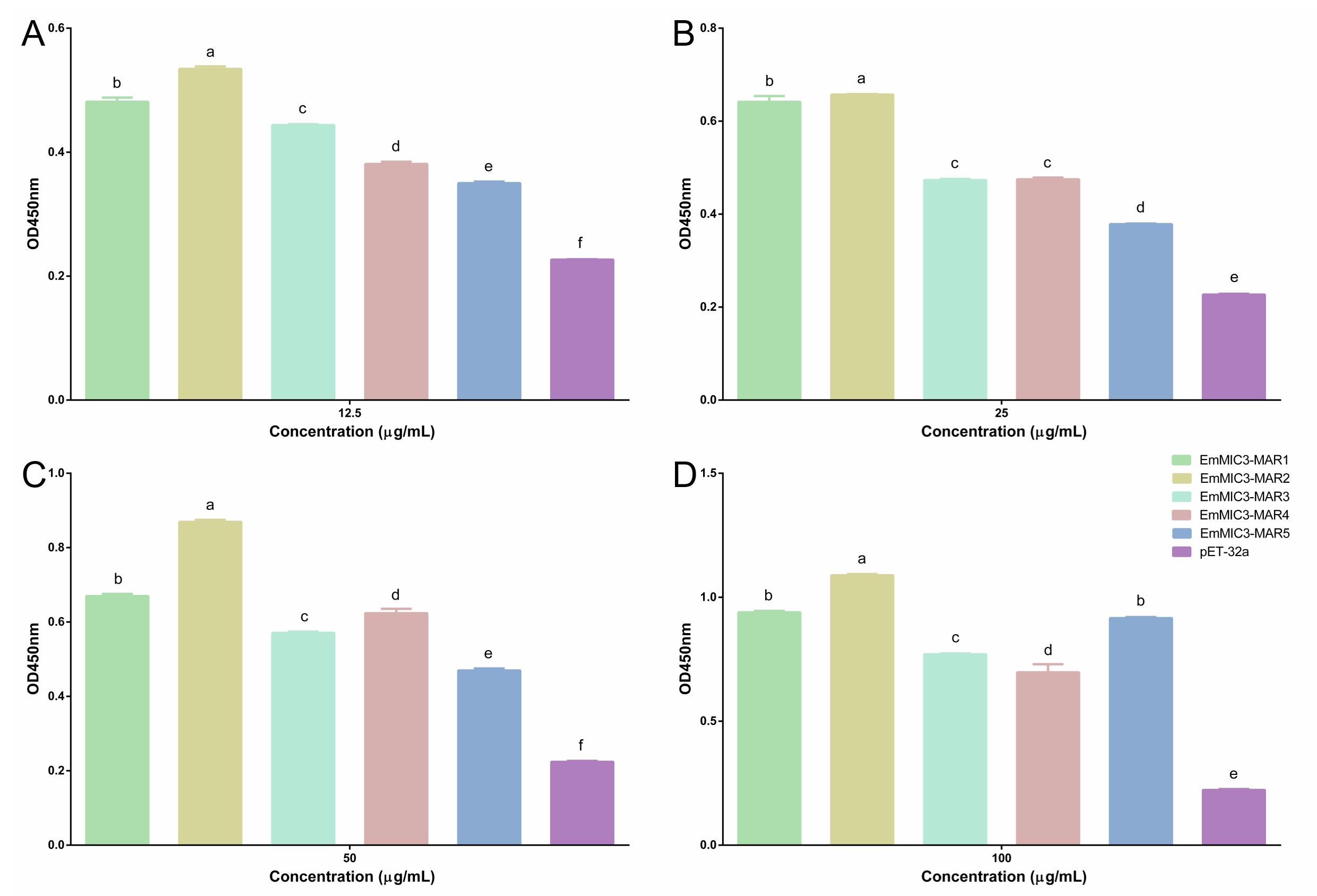

Supplement: Supplementary File 2 — Binding of EmMIC3-MARs to fixed mid intestinal epithelial cells determined by ELISA. The mid intestinal epithelial cells were isolated and cultivated from 2-week-old chickens. The chicken mid intestinal epithelial cells were co-cultured respectively with the recombinant EmMIC3-MARs. The final concentration of every protein were 12.5 μg/mL (A), 25 μg/mL (B), 50 μg/mL (C), and 100 μg/mL (D). Among different MARs, the significant difference within the group was shown by different letters (p < 0.05). [file Image_2.tif]

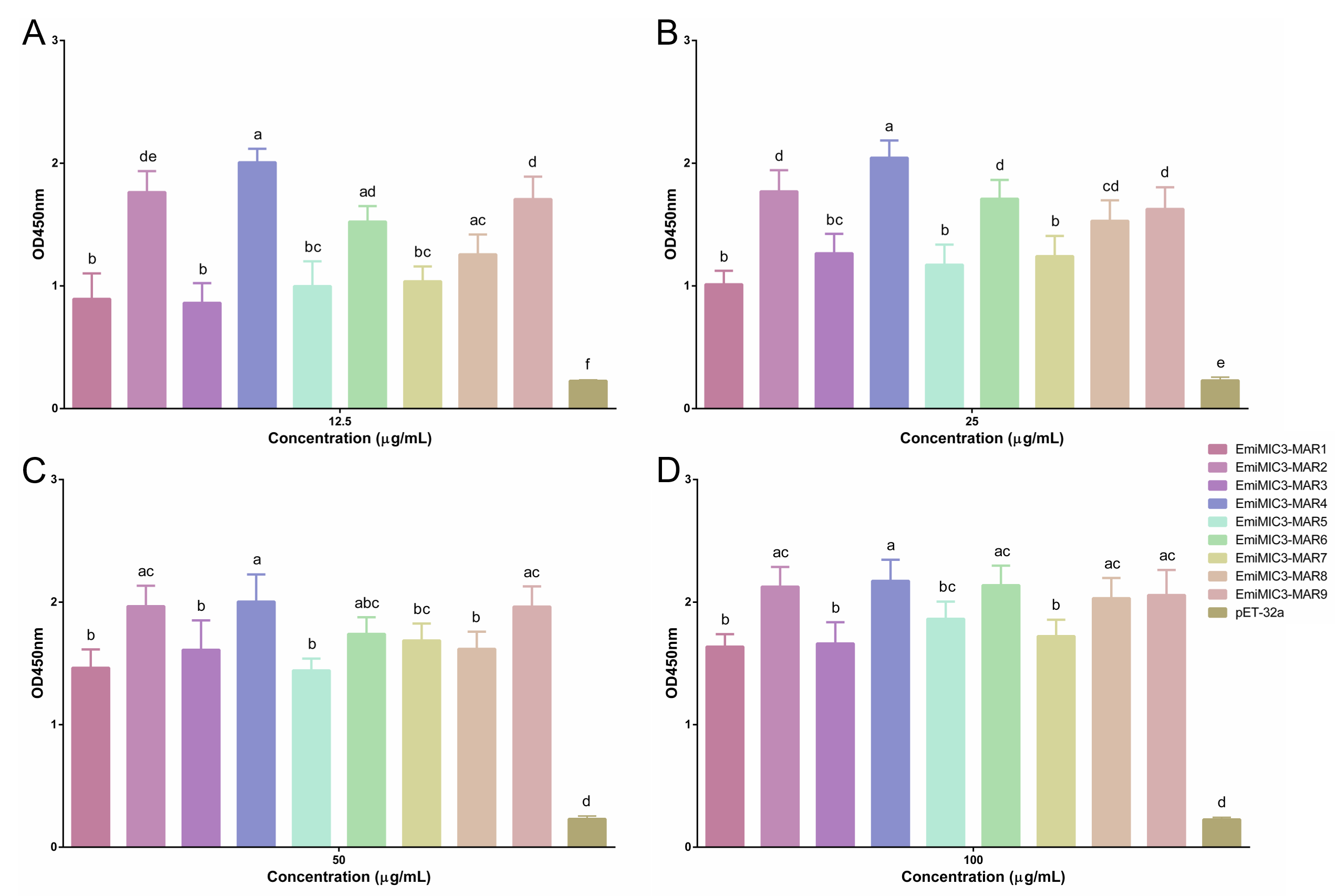

Supplement: Supplementary File 3 — Binding of EmiMIC3-MARs to fixed lower intestinal epithelial cells determined by ELISA. The lower intestinal epithelial cells were isolated and cultivated from 2-week-old chickens. The chicken lower intestinal epithelial cells were co-cultured respectively with the recombinant EmiMIC3-MARs. The final concentration of every protein were 12.5 μg/mL (A), 25 μg/mL (B), 50 μg/mL (C), and 100 μg/mL (D). Among different MARs, the significant difference within the group was shown by different letters (p < 0.05). [file Image_3.tif]
