# Supplementary material for: The microneme adhesive repeat domain of MIC3 protein determined the site specificity of Eimeria acervulina, Eimeria maxima, and Eimeria mitis
Source: Front Immunol. 2023 Nov 8;14:1291379. doi: 10.3389/fimmu.2023.1291379 (PMC10663340; doi:10.3389/fimmu.2023.1291379)
Supplement: Supplementary file 4 [file Table_1.docx]

**Table S1 oligonucleotide primer sequences for RACE**

| Primer name | Sequence（5’-3’） |
| --- | --- |
| 5'-CDS Primer A | （T）_25_V N |
| 3'-CDS Primer A | AAGCAGTGGTATCAACGCAGAGTAC（T）_30_V N |
| SMARTer II A | AAGCAGTGGTATCAACGCAGAGTACXXXXX |
| UPM | CTAATACGACTCACTATAGGGCAAGCAGTGGTATCAACGCAGAGT |
| GSP1 | CTGGCTGTTGCGTGACAGGTAAGTTTT |
| GSP2 | GGCATGTCCGGTGAGGGTTGT |
| NUPM | AAGCAGTGGTATCAACGCAGAGT |
| NGSP1 | TTGCTGTTGACATTGTTGCTTGCTCC |
| NGSP2 | CTCGGGATGCTGGGCTAAGGT |
