# Supplementary material for: The microneme adhesive repeat domain of MIC3 protein determined the site specificity of Eimeria acervulina, Eimeria maxima, and Eimeria mitis
Source: Front Immunol. 2023 Nov 8;14:1291379. doi: 10.3389/fimmu.2023.1291379 (PMC10663340; doi:10.3389/fimmu.2023.1291379)
Supplement: Supplementary file 5 [file Table_2.docx]

**Table S2 Oligonucleotide primer sequences used for PCR**

| Name | Sequences (5’→3’) | Restriction enzyme |
| --- | --- | --- |
| EaMIC2-F | CCGGAATTCATGGGGGATGAACCATCCTC | *Eco*RⅠ |
| EaMIC2-R | CCCAAGCTTTTCTATTTCTATGAGCTGGCGG | *Hind* III |
| EaMIC3-F | CGCGGATCCATGCCTGTATATGCGAGATACGACG | *Bam*H I |
| EaMIC3-R | CCGCTCGAGAACACACTGTCTCGTCATTGCCG | *Xho* I |
| EaMIC5-F | CCGGAATTCGAGCTCTGCTACAAACACCCG | *Eco*R I |
| EaMIC5-R | CCCAAGCTTGCCGTTATTAAATGCATGCG | *Hin*d III |
| EmMIC2-F | CCG*GAATTC*ATGGCTCGCGCCCTTTCAT | *Eco*RⅠ |
| EmMIC2-R | CCC*CTCGAG*CTAGGAGCTGACCGATGTTGTGG | *Xho* I |
| EmMIC3-F | *GATATC*ATGAAGGTGCCTTCGGCT | *EcoR* V |
| EmMIC3-R | *CTCGAG*CTAGAACCCGTCCAAGG | *Xho* I |
| EmAMA1-F | *AAGCTT*CCTCTCAGCGATTTCATGG | *Hind* III |
| EmAMA1-R | *CTCGAG*CTAGTAATCTTGGTCAACTAACACG | *Xho* I |
| EmiMIC2-F | GCTGATATCGGATCCGAATTCATGTGCCCACCCGGCACGATACG | *Eco*RⅠ |
| EmiMIC2-R | TCGAGTGCGGCCGCAAGCTTTTACGAAGCCCACATCTCTGTCTGC | *Hin*d III |
| EmiMIC3-F | GCCATGGCTGATATCGGATCCATGAAGGTGCCTTCGGCCATCG | *Bam*H I |
| EmiMIC3-R | TGGTGGTGGTGGTGCTCGAGTCAATGCTGATGCACGTGGACC | *Xho* I |
| EmiAMA1-F | GCTGATATCGGATCCGAATTCATGGTGATAGGCTTGGCT | *Eco*RⅠ |
| EmiAMA1-R | TCGAGTGCGGCCGCAAGCTTTTAGTAGTCTTCATCTACT | *Hin*d III |
| EmiEtmic-2/7h-F | GCTGATATCGGATCCGAATTCATGGCTCGCGCTTTTTCCGTAAT | *Eco*R Ⅰ |
| EmiEtmic-2/7h-R | TCGAGTGCGGCCGCAAGCTTCTATACGTCCTCCGACGCATCCGAA | *Hin*d III |
