# Supplementary material for: The microneme adhesive repeat domain of MIC3 protein determined the site specificity of Eimeria acervulina, Eimeria maxima, and Eimeria mitis
Source: Front Immunol. 2023 Nov 8;14:1291379. doi: 10.3389/fimmu.2023.1291379 (PMC10663340; doi:10.3389/fimmu.2023.1291379)
Supplement: Supplementary file 6 [file Table_3.docx]

**Table S3 Oligonucleotide primer sequences used for MARs amplication**

| Accession number | Repeat | Residue | Recombinant expression | Sequencs(5’-3’) | Restriction enzyme |
| --- | --- | --- | --- | --- | --- |
| AMN15064.1 | EaMIC3-MAR1 | 3-76 | recEaMIC3-MAR1 | CGCGGATCCGTATATGCGAGATACGACGGAAGAA | *Bam*H I |
|  |  |  |  | CCGCTCGAGTCAACTGCAATATTCGGGCTTCAAT | *Xho* I |
|  | EaMIC3-MAR2 | 87-184 | recEaMIC3-MAR2 | CGCGGATCCGAAGCTTTGGACAGAAAGTGCG | *Bam*H I |
|  |  |  |  | CCGCTCGAGTCAGCAGTAGAAATCTCTGTTGCTGGTC | *Xho* I |
|  | EaMIC3-MAR3 | 221-318 | recEaMIC3-MAR3 | CGCGGATCCTTGGACAAGGTTTGCGCTGAG | *Bam*H I |
|  |  |  |  | ATTTGCGGCCGCAAGGTGATTCTGATGCACGTCCG | *Not* I |
|  | EaMIC3-MAR4 | 352-449 | recEaMIC3-MAR4 | CGCGGATCCCTGCAGGAGGTCTTGGACAACC | *Bam*H I |
|  |  |  |  | CCGCTCGAGTCAAGGTGATTCCGATGTGCGTCC | *Xho* I |
|  | EaMIC3-MAR5 | 485-582 | recEaMIC3-MAR5 | CGCGGATCCCACTTCGTGGACGAATTTTGTCTG | *Bam*H I |
|  |  |  |  | CCGCTCGAGTCAAGGATTGCAGAATTCGTCTAGCTTC | *Xho* I |
|  | EaMIC3-MAR6 | 618-715 | recEaMIC3-MAR6 | CGCGGATCCGCTTTGGTGGACGGGTTTTGT | *Bam*H I |
|  |  |  |  | CCGCTCGAGTCAGCACCGTCCAGCTTTCTCTGTG | *Xho* I |
|  | EaMIC3-MAR7 | 772-852 | recEaMIC3-MAR7 | CGCGGATCCATGGTGTTCTTTGCAAAGCAGTGT | *Bam*H I |
|  |  |  |  | CCGCTCGAGTCAGCAAGTGCCGCTCTTCAACTCT | *Xho* I |
| AOY42085.1 | EmMIC3-MAR1 | 51-144 | recEmMIC3-MAR1 | CGAGCTCCTGGACGACTTGTGC | *Sac* Ⅰ |
|  |  |  |  | ATTTGCGGCCGCTTTCAATGCATCCACCTGG | *Not* Ⅰ |
|  | EmMIC3-MAR2 | 159-248 | recEmMIC3-MAR2 | CGAGCTCTTGGACAGAAAGTGCGCTGCTTTT | *Sac* Ⅰ |
|  |  |  |  | ATTTGCGGCCGCAATTACTGAGGAGATCTCGTTTCCGCGAGA | *Not* Ⅰ |
|  | EmMIC3-MAR3 | 278-371 | recEmMIC3-MAR3 | CGAGCTCTTGGACGGGTTCTGCGCTGAG | *Sac* Ⅰ |
|  |  |  |  | ATTTGCGGCCGCCTTGTTTGCAGCAAGCGCTGACCTT | *Not* Ⅰ |
|  | EmMIC3-MAR4 | 412-505 | recEmMIC3-MAR4 | CGAGCTCTTGGACGGGTT | *Sac* Ⅰ |
|  |  |  |  | ATTTGCGGCCGCTTTCTTTTTCTGAATGAGTT | *Not* Ⅰ |
|  | EmMIC3-MAR5 | 558-639 | recEmMIC3-MAR5 | CGAGCTCCTTGATTCCCAGTGTAT | *Sac* Ⅰ |
|  |  |  |  | ATTTGCGGCCGCCTGTTGGGCCATTGC | *Not* Ⅰ |
| AXC32915.1 | EmiMIC3-MAR1 | 49-142 | recEmiMIC3-MAR1 | GCCATGGCTGATATCGGATCCCTGGACGAGTTTTGCCAAAG | *Bam*H I |
|  |  |  |  | TGGTGGTGGTGGTGCTCGAGTCTTCAGTGAATCAACTTTC | *Xho* I |
|  | EmiMIC3-MAR2 | 157-250 | recEmiMIC3-MAR2 | GCCATGGCTGATATCGGATCCTTGGACAGAAAGTGTATGA | *Bam*H I |
|  |  |  |  | TGGTGGTGGTGGTGCTCGAGTCTTGTCTGCGGCAATAACTGA | *Xho* I |
|  | EmiMIC3-MAR3 | 315-408 | recEmiMIC3-MAR3 | GCCATGGCTGATATCGGATCCTTGGACCAGATGTGTGTTGAG | *Bam*H I |
|  |  |  |  | TGGTGGTGGTGGTGCTCGAGTCTTGTTTGCTTCAATCGCGT | *Xho* I |
|  | EmiMIC3-MAR4 | 456-546 | recEmiMIC3-MAR4 | GCCATGGCTGATATCGGATCCTTGGACAACTTGTGCATTC | *Bam*H I |
|  |  |  |  | TGGTGGTGGTGGTGCTCGAGTTGCGTTGATCGCTGACTGT | *Xho* I |
|  | EmiMIC3-MAR5 | 569-662 | recEmiMIC3-MAR5 | GCCATGGCTGATATCGGATCCCTGGACGAGTTTTGCCAAAG | *Bam*H I |
|  |  |  |  | TGGTGGTGGTGGTGCTCGAGTCTTCAGTGAATCAACTTTC | *Xho* I |
|  | EmiMIC3-MAR6 | 677-767 | recEmiMIC3-MAR6 | GCCATGGCTGATATCGGATCCCTGGACGAGTTTTGCCAAAG | *Bam*H I |
|  |  |  |  | TGGTGGTGGTGGTGCTCGAGTCTTCAGTGAATCAACTTTC | *Xho* I |
|  | EmiMIC3-MAR7 | 789-882 | recEmiMIC3-MAR7 | GCCATGGCTGATATCGGATCCCTGGACGAGTTTTGCCAAAG | *Bam*H I |
|  |  |  |  | TGGTGGTGGTGGTGCTCGAGTCTTCAGTGAATCAACTTTC | *Xho* I |
|  | EmiMIC3-MAR8 | 915-1008 | recEmiMIC3-MAR8 | GCCATGGCTGATATCGGATCCCTGGACGAGTTTTGCCAAAG | *Bam*H I |
|  |  |  |  | TGGTGGTGGTGGTGCTCGAGTCTTCAGTGAATCAACTTTC | *Xho* I |
|  | EmiMIC3-MAR9 | 1042-1110 | recEmiMIC3-MAR9 | GCCATGGCTGATATCGGATCCCTGGACGAGTTTTGCCAAAG | *Bam*H I |
|  |  |  |  | TGGTGGTGGTGGTGCTCGAGTCTTCAGTGAATCAACTTTC | *Xho* I |
