# Supplementary material for: The microneme adhesive repeat domain of MIC3 protein determined the site specificity of Eimeria acervulina, Eimeria maxima, and Eimeria mitis
Source: Front Immunol. 2023 Nov 8;14:1291379. doi: 10.3389/fimmu.2023.1291379 (PMC10663340; doi:10.3389/fimmu.2023.1291379)
Supplement: Supplementary file 7 [file Table_4.docx]

**Table S4 Oligonucleotide primer sequences used for PCR**

| Name | Sequences (5’→3’) | Restriction enzyme |
| --- | --- | --- |
| EtMAR1bcde-F | CGCGGATCCATGTTGAAATTACGCACACAAGAGTTCTG | *Bam*H I |
| EtMAR1bcde-R | CCGCTCGAGCTAGTCTGACGCTCCCATTTGATTG | *Xho* I |
| EtMAR1cde-F | ATTTGCGGCCGCATACCTGAAGGGACACCTGAGGA | *Not* I |
| EtMAR1cde-R | CCGCTCGAGCTACAATGTGGCCCTCTCCCC | *Xho* I |
| EtMIC3-MAR1b-F | CGCGGATCCGCCCCAGCGCCAACGCTT | *Bam*H I |
| EtMIC3-MAR1b-R | ATTTGCGGCCGCATCAATCAGTGCAGTAGTTGCTTTTGTCGCT | *Not* I |
| EaMIC3-MAR12-F | GGAAGATCTGGTATATGCGAGATACGACGGAAGAA | *Bgl* Ⅱ |
| EaMIC3-MAR12-R | CGCGGATCCGCAGTAGAAATCTCTGTTGCTGGTC | *Bam*H I |
| EaMIC3-MAR4-7-F | ATTTGCGGCCGCCTGCAGGAGGTCTTGGACAACC | *Not* I |
| EaMIC3-MAR4-7-R | CCGCTCGAGTCAGCAAGTGCCGCTCTTCAACTCT | *Xho* I |
